# Residual-hybrid dynamic response-surface/machine-learning framework for predicting malachite green removal and biodegradation by a bacterial consortium

**DOI:** 10.1186/s13036-026-00663-8

**Published:** 2026-04-02

**Authors:** Ebtehag A. E. Sakr, Marwa S. Hassan, Mervat El‑Sedik, Manal M. Rekaby, Ghada M. El-Sayed

**Affiliations:** 1 Botany Department, Faculty of Women for Arts, Science and Education, Ain Shams University, Cairo, Egypt; 2 Systems and Information Department, Engineering Research Institute, New and Renewable Energy, National Research Centre (NRC), 33 El- Behouth St, Dokki, Giza 12622 Egypt; 3 Dyeing, Printing and Textile Auxiliaries Department, Textile Research and Technology Institute, National Research Centre, 33 EL Buhouth St., Dokki, Giza 12622 Egypt; 4 Microbial Genetics Department, Biotechnology Research Institute, National Research Centre, 33 El-Behouth St, Dokki, Giza 12622 Egypt

**Keywords:** Consortium, Malachite green, Biodegradation, Response surface methodology (RSM), Machine learning, Hybrid modelling

## Abstract

**Background:**

The discharge of dye-containing effluents is a global environmental concern, particularly for synthetic cationic dyes such as malachite green (MG). This study aimed to develop and experimentally validate a hybrid dynamic response-surface/machine-learning framework, coupled with an optimized bacterial consortium, for predicting and enhancing MG removal and elucidating its biodegradation pathway.

**Results:**

A four-strain comprising *Azotobacter chroococcum*,* Azotobacter salinestris*,* Stenotrophomonas maltophilia*, and *Sphingomonas kyeonggiensis* was first optimized by mixture design, where an appropriate proportion of *A. chroococcum* and *S. maltophilia* achieved 83.51% MG decolorization. A Box–Behnken design (BBD) was then applied to optimize initial MG concentration, inoculum size, and incubation time; the response-surface model predicted a maximum removal of 96.56% at 100 mg/L, 7% inoculum, and 72 h, in good agreement with the experimental data. The resulting RSM-based dynamic response-surface model used as a baseline, and residual machine-learning regressors were trained on experimentally derived residuals (experimental MG removal minus baseline prediction). Among standalone learners, CatBoost provided the best performance on the exploratory 80/20 train–test split, whereas under leave-one-out cross-validation, the hybrid residual model (dynamic response-surface + Ridge regression) emerged as the most reliable predictor, yielding closer agreement with the experimental MG removal than the baseline model. Biodegradation of MG by the optimized consortium was experimentally validated by Fourier-transform infrared (FTIR) spectroscopy, liquid chromatography–mass spectrometry (LC–MS) and UV–visible spectroscopy, which confirmed disruption of the chromophoric structure and formation of less toxic metabolites. Toxicity assay indicated that the degradation products were non-toxic towards the tested pathogenic bacteria. ISSR molecular typing revealed that primer ISSR-12 exhibited strong discriminatory power, generating nine strain-specific bands, whereas primer ISSR-1 produced a higher proportion of monomorphic bands.

**Conclusions:**

This work introduces a small-sample, hybrid dynamic response-surface/machine-learning framework, experimentally validated against MG biodegradation experiments, which integrates an RSM-based baseline as an explicit feature in residual learning. Under leave-one-out cross-validation (*N* = 17), the RSM baseline achieved R² = 0.988 and RMSE = 2.62% MG removal, while the best hybrid residual model (dynamic response-surface + Ridge regression) yielded R² = 0.987 and RMSE = 2.79%, confirming the robustness of the proposed framework.

**Supplementary Information:**

The online version contains supplementary material available at 10.1186/s13036-026-00663-8.

## Introduction

Sustainable management of wastewater from the textile sector remains one of the most pressing environmental challenges for the current administration. A significant portion of textile effluents are discharged into the environment each year without adequate pretreatment, which negatively affects both plant productivity and human health [1]. Malachite Green (MG) is a synthetic cationic dye widely used to color materials such as wood, cotton, and nylon, and has also been extensively applied in aquaculture to control protozoal and fungal infections [2]. However, MG is highly toxic and has been associated with mutagenic, teratogenic, and carcinogenic effects, leading to its regulatory ban in many countries [2]. Despite these restrictions, its use persists in several regions due to its low cost and high efficacy [2]. Due to its persistence in aquatic and industrial environments, there is an urgent need for degradative and detoxification strategies [2, 3]. Globally, industrial assessments estimate that approximately 700,000–1,000,000 tons of synthetic dyes are produced annually, of which about 10–15% are discharged into wastewater streams during manufacturing and dyeing operations [4]. MG constitutes a substantial fraction of these emissions, making the development of cost-effective and efficient treatment processes a continuing challenge [3].

Traditional physicochemical methods for color removal are often inconvenient due to the high-water solubility of dyes, elevated operating costs, and the generation of secondary pollution [5]. Biological decolorization offers a more sustainable alternative, enabling the treatment of dye effluents from multiple industrial sectors [6]. In microbial consortia, synergistic interactions take place when microorganisms cooperate to provide an effect that is greater than the sum of their individual actions [7, 8]. In microbial consortia for dye bioremediation, these interactions are crucial because they improve environmental tolerance, increase metabolic capacity, improve degradation efficiency, and decrease the generation of hazardous intermediates. Thus, compared to individual strains, microbial consortia provide a more efficient and adaptable method for degrading environmental pollutants, making them useful instruments for enhanced bioremediation processes [9–11]. Leveraging such bacterial synergism to develop eco-friendly strategies for decomposing textile dye effluents has therefore become a growing trend [12].

To systematically exploit these interactions, mixture design (MD) has been widely employed to study the behavior of mixed cultures and compare the decolorization efficiency of single versus combined strains [13]. Regression analysis is commonly used to optimize the consortium formulation [13]. In parallel, response surface methodology (RSM), including Box–Behnken design (BBD), allows efficient optimization of process conditions with a limited number of experiments [14]. Using BBD, researchers can quantify both the individual and interactive effects of process variables and identify operating conditions that maximize decolorization efficiency [15, 16].

From a modelling standpoint, conventional approaches for dye removal — such as kinetic or mechanistic formulation can capture the essential dynamics of the system, but they may struggle to represent complex nonlinear behavior or unmodelled interactions among process variables. In contrast, machine-learning (ML) techniques (Table S1) (e.g., regression and boosting algorithms) are highly flexible and can approximate complex relationships directly from data, but they typically require larger datasets and often lack physical interpretability [17]. In this work, rather than developing a full mechanistic kinetic model, we adopt an empirical dynamic response-surface (RSM) baseline (hereafter referred to as the dynamic response-surface model) generated with Design-Expert^®^ v7.0 to describe the time-dependent and design-dependent behavior of MG decolorization within the experimental domain. Machine-learning models are then trained on the residuals between the experimental MG removal and this dynamic response-surface baseline. This hybrid strategy combines the structured, design-based insight of the RSM baseline with the flexibility of data-driven ML models, while preserving a clear physical link to the underlying experimental design space.

In response to these limitations, hybrid and physics-informed modeling frameworks—which integrate a structured baseline model with data-driven residual correction—have gained increasing attention across many scientific and engineering fields [18, 19]. To improve generalization and regularize learning, for example, physics-informed neural networks (PINNs) incorporate governing equations directly into deep architectures [20]. First-principles or design-based models have been coupled with machine learning corrections for reactor performance and thermophysical properties in chemical and process engineering using science-guided and residual-hybrid approaches [21, 22]. Residual-hybrid ensembles have also been applied to time-series forecasting, where ML models learn directly from model-error structures to improve predictive accuracy [18, 19]. In fluid dynamics, frequency–residual deep-learning models have been developed for unsteady flow-field reconstruction and turbulence-model correction [20, 21]. Collectively, these studies demonstrate the value of combining mechanistic insight with data-driven flexibility.

**Fig. 1:**
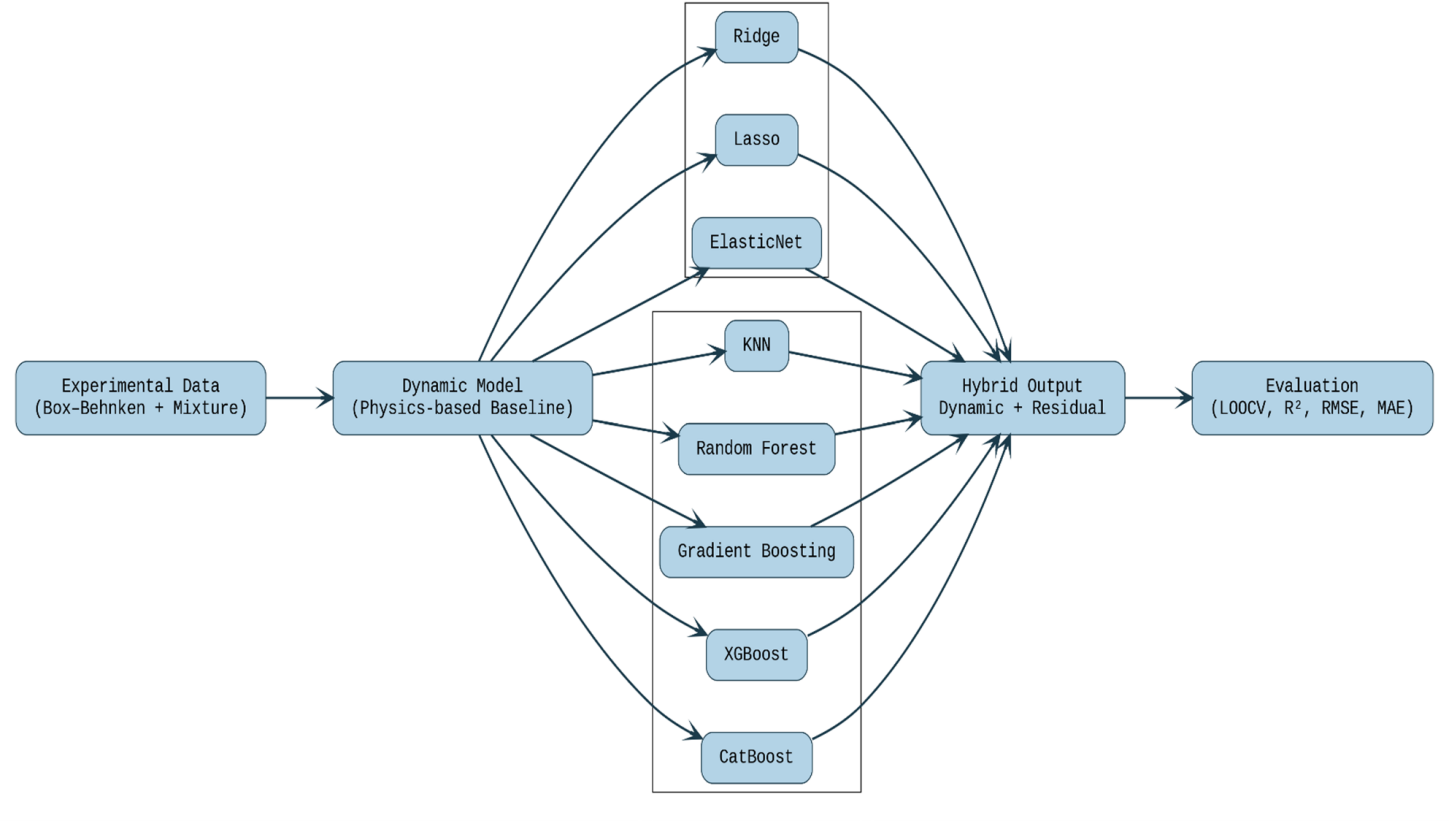
Workflow of the proposed hybrid modeling framework. The *“Dynamic Model”* represents an empirical RSM-based dynamic response-surface baseline generated using *Design-Expert*^*®*^* v7.0.* Residual machine-learning regressors are trained on the difference between experimental MG-removal values and this baseline prediction, and the hybrid output *(Dynamic + Residual)* is evaluated using *LOOCV (R²*,* RMSE*,* MAE)*

In this work, we adopt a similar hybrid philosophy, tailoring it to small-sample environmental biotechnology. Instead of a full mechanistic kinetic description, we employ an RSM-based dynamic response-surface model (generated using Design-Expert^®^ v7.0) as an interpretable and data-efficient structured baseline over the experimental design space. Residual machine-learning regressors are then trained on experimentally derived residuals (experimental MG removal minus RSM baseline prediction) to correct systematic errors and improve prediction accuracy under small-sample constraints. This hybrid dynamic response-surface/machine-learning framework, therefore, integrates the strengths of response-surface modeling with residual learning for dye-removal applications as presented in Fig. 1.

Furthermore, recent advances show that hybrid residual modeling can significantly outperform purely data-driven methods in systems with limited datasets. Physics-informed and residual deep-learning models have successfully corrected deficiencies in flow solvers and first-principles models, for example, in unsteady flow reconstruction and ionic-liquid property prediction [21, 22]. By contrast, MG-removal studies rely predominantly on black-box Artificial Neural Network Genetic Algorithms (ANN–GA) or Artificial Neural Network Response surface Modeling (ANN–RSM) models without a structured physical baseline or explicit residual correction [23–25]. Hence, to the best of our knowledge, no prior study has proposed a residual-hybrid, RSM-guided machine-learning framework for MG removal or wastewater treatment—establishing the novelty of the present work.

Inter-simple sequence repeat polymerase chain reaction (ISSR-PCR) is a robust and cost-effective technique for studying genetic diversity. ISSR-PCR provides a rapid molecular marker system for evaluating genetic similarity among species [26]. The method employs primers that target variable regions between adjacent microsatellites, which are evolutionarily conserved [27], making ISSR-PCR a powerful tool for exploring genetic variation and elucidating phylogenetic relationships among bacterial strains.

Nevertheless, relatively few studies have investigated the use of *Stenotrophomonas maltophilia* for MG degradation, and reports on MG biodegradation by *Azotobacter chroococcum*, *Azotobacter salinestris* and *Sphingomonas kyeonggiensis* remain scarce. Building on these gaps, the present study has three main objectives: **(i)** to formulate and optimize a four-strain bacterial consortium—comprising *Azotobacter chroococcum* 6FE (PQ783059), *Azotobacter salinestris* 9FE (PQ783060), *Stenotrophomonas maltophilia* strain 20-salinity (OK340221) and *Sphingomonas kyeonggiensis*—for MG decolorization using mixture design and BBD-based RSM; **(ii)** to develop and experimentally validate a small-sample hybrid dynamic response-surface/machine-learning residual framework for predicting MG removal; and **(iii)** to elucidate the MG biodegradation pathway, assess the toxicity of the resulting metabolites, and investigate the genetic diversity of the bacterial strains using ISSR-PCR.

## Material and methods

### Dye, media, and chemicals

For this investigation, MG dye was provided by the Botany Department of the Faculty of Women at Ain Shams University, Egypt. After being made at a concentration of 1000 mg/L (w/v), the stock dye solution was autoclaved for 15 min at 121 °C and then stored at 4 °C. The necessary concentrations of dye solutions were obtained by successive dilutions. Nutrient agar (NA) and nutrient broth (NB) were utilized. NB was composed of 5 gL^− 1^ peptone, 5 gL^− 1^ NaCl, 3 gL^− 1^ beef extract, and 2 gL^− 1^ yeast extract. The highest purity and analytical grade of chemicals were utilized in this investigation.

### Bacterial strains and culture conditions

*Azotobacter chroococcum* 6FE (PQ783059), *Azotobacter salinestris* 9FE (PQ783060) [28], *Stenotrophomonas maltophilia* strain 20-salinity (OK340221), and *Sphingomonas kyeonggiensis* strain 23-salinty (OK340222) were used in this study. Before being used, these bacterial strains were kept in cryovials with 20% (v/v) glycerol solution at -80 °C after being previously isolated from soil and identified by 16 S rRNA gene sequence analysis. The strains were reactivated on NA plates at 37 °C for 24 h. An earlier test of antagonism was conducted to demonstrate the synergy of strains (under publication) to facilitate the development of a consortium for MG biodegradation. The NB medium was used to culture and maintain the bacterial strains at 37 °C. The bacteria were taken after being incubated for 24 to 48 h, and they were then centrifuged at 10,000 rpm. The supernatant was disposed of, and the resulting bacterial pellets were gathered. Following the harvesting process, the biomass was cleaned and dissolved in a 0.8% NaCl solution to develop a suspension. The decolorization tests were then conducted using the produced suspension.

### Optimization of MG decolorization by bacterial consortium

#### Mixture design (MD)

The D-optimal mixture design was used to investigate the relation between the proportions of independent components (various strains) and the yield of MG decolorization: A; *Azotobacter chroococcum*, B; *Azotobacter salinestris*, C; *Stenotrophomonas maltophilia*, and D; *Sphingomonas kyeonggiensis*. Using this technique, the ideal ratio of ingredients for the consortium formulation in relation to the response (MG decolorization) was ascertained. To prepare the consortium, the D-optimal mixture design parameters were established using Design-Expert Version 7.0 software in different ratios between 0% and 100% (Table 1). Twenty consortium formulations with varying proportions of each strain were produced by these four factors (Table 1). 4% of the prepared consortium was seeded into the NB containing 100 mg/L dye and incubated at 37 °C for 24 h under static conditions. Decolorization studies were carried out in accordance with the ratio specified by the experimental design Abiotic control was maintained without inoculation. The bacteria were centrifuged at 8000 rpm for 10 min to measure the percentage of decolorization. The following formula [29] was used to monitor the MG decolorization using UV-visible spectroscopy (λ max = 618 nm). Decolorization of MG (%) = [(initial absorbance– observed absorbance)/initial absorbance] × 100.

**Table 1:** Mixture-design matrix and experimental MG decolorization (%) for the four-strain consortium, together with predictions from the RSM-based dynamic response-surface model (Design-Expert^®^ v7.0)

Run	Component 1	Component 2	Component 3	Component 4	MG decolorization (%)	
	A: X1	B: X2	C: X3	D: X4	Experimental (actual)	RSM-based dynamic baseline (Predicted)
1	0	100	0	0	33.01	33.03
2	12.406	12.327	62.4	12.867	82.22	81.67
3	100	0	0	0	68.63	68.65
4	62.593	12.346	12.574	12.487	63.01	62.64
5	12.601	62.745	12.128	12.527	59.17	58.82
6	0	50	50	0	74.69	74.81
7	0	49.999	0	50.001	33.54	33.65
8	50	50	0	0	77.39	77.47
9	0	0	50.001	49.999	53.13	54.83
10	0	100	0	0	33.01	33.03
11	0	0	50.001	49.999	56.41	54.83
12	49.999	0	50.001	0	83.51	83.63
13	13.399	13.675	9.149	63.777	47.99	47.55
14	0	0	0	100	28.53	29.52
15	100	0	0	0	68.63	68.65
16	50	0	0	50	64.28	64.39
17	0	0	100	0	66.35	67.68
18	0	0	100	0	68.91	67.68
19	0	0	0	100	30.45	29.52
20	25.25	24.752	24.729	25.269	66.03	66.87

**MG**: malachite green

**X1**: *A. chroococcum 6FE*; **X2**: *A. salinestris 9FE*; **X3**: *S. maltophilia*; **X4**: *S. kyeonggiensis*

The empirical model was constructed using MG decolorization data, and its adequacy was assessed through various statistical parameters, including the coefficient of determination (R²), adjusted R², predicted R², coefficient of variation (C.V.), predicted residual sum of squares (PRESS), lack-of-fit tests, and regression statistics such as the p-value and F-value. These metrics facilitated the selection of the best-fitting model for the process. The model equation derived from the statistical analysis included coefficients, where positive values indicated a beneficial effect on the response, while negative values indicated a detrimental effect. Also, regression coefficients with 95% confidence intervals was determined. To enhance understanding, three-dimensional response surface graphs and contour plots were generated to visualize the effects of variables and their interactions on the response. This methodology is standard in BBD analysis, optimizing experimental conditions and effectively interpreting the impacts of variables.

#### Box Behnken design (BBD)

The study utilized the BBD to optimize parameters and evaluate interactions affecting the removal of MG dye from nutrient broth media by a microbial consortium. The variables tested included initial dye concentration (100–700 mg/L), inoculum size (4.0–10.0%), and incubation time (24–72 h), analyzed across 17 experimental runs at three levels (-1, 0, + 1; Table 3). The response was measured as the percentage of MG decolorization. Model validation was confirmed by comparing observed and predicted results and also regression coefficients with 95% confidence intervals. Experimental design and data analysis were conducted using Design-Expert software V7.0, which facilitated the generation of polynomial models to describe the effects of the variables and their interactions on MG removal efficiency. This approach effectively optimized MG decolorization conditions under controlled variables.

**Table 2:** Analysis of variance (ANOVA) for malachite green (MG) removal efficiency (%)

Source	Sum of Squares	df	Mean Square	F-Value	*P*-value	
Model	6026.16	13	463.55	231.19	< 0.0001	significant
Linear Mixture	4415.63	3	1471.88	734.09	< 0.0001	
AB	568.79	1	568.79	283.68	< 0.0001	
AC	192.26	1	192.26	95.89	< 0.0001	
AD	188.09	1	188.09	93.81	< 0.0001	
BC	480.24	1	480.24	239.52	< 0.0001	
BD	4.54	1	4.54	2.26	0.1832	
CD	51.74	1	51.74	25.81	0.0023	
ABC	24.18	1	24.18	12.06	0.0133	
ABD	114.69	1	114.69	57.2	0.0003	
ACD	5	1	5	2.49	0.1655	
BCD	185.99	1	185.99	92.76	< 0.0001	
Residual	12.03	6	2.01			
Lack of Fit	1.53	1	1.53	0.73	0.4322	NS
Pure Error	10.5	5	2.1			
Cor Total	6038.19	19				

**Model summary**: Std. Dev. = 1.42; Mean = 57.94; C.V.% = 2.44; PRESS = 1175.53; R² = 0.998; Adj. R² = 0.9937; Pred. R² = 0.8053; Adeq. Precision = 45.675

**Significance criterion**: For Prob > F, values < 0.050 indicate statistically significant effects, whereas values > 0.100 indicate non-significant (**NS**) effects

**A**: *A. chroococcum* 6FE; **B**: *A. salinestris* 9FE; **C**: *S. maltophilia*; **D**: *S. kyeonggiensis*

**Table 3:** Three-factor BBD matrix for MG dye removal, with experimental results and RSM-based dynamic response-surface baseline predictions

Run	F1 A: Initial dye concentration (mg/L)	F 2 B: Amount of consortia inoculum (v/v %)	F 3 C: Incubation time (h)	Response MG decolorization (%)	
				Experimental )actual)	RSM-based dynamic baseline (Predicted)
1	700	10	48	53.62	51.11
2	400	7	48	89.06	83.10
3	400	4	72	88.42	87.15
4	100	7	24	88.08	87.42
5	400	10	72	96.05	97.91
6	700	7	72	46.66	47.31
7	400	10	24	78.64	79.90
8	400	7	48	83.2	83.10
9	700	4	48	14.39	15.00
10	100	7	72	97.81	96.56
11	400	7	48	86.16	83.10
12	400	7	48	79.73	83.10
13	400	7	48	77.35	83.10
14	400	4	24	72.76	70.90
15	700	7	24	20.95	22.20
16	100	10	48	82.73	82.12
17	100	4	48	95.95	98.46

Note: “Dynamic model” denotes the empirical time-dependent response-surface baseline generated in Design-Expert 7.0

### Machine learning and hybrid residual modeling framework

Machine-learning analysis was implemented to (i) benchmark purely data-driven models against the response-surface-based dynamic model and (ii) construct a hybrid residual framework that corrects the mechanistic predictions using flexible regressors.

### Dataset and input variables

Descriptive statistics, distribution plots, and correlation heatmaps were computed for both designs using Python/Matplotlib/Seaborn.” The dataset consists of N = 17 experimental runs obtained from the MD and BBD. Given the limited sample size, we employed a Leave-One-Out Cross-Validation (LOOCV) [30] strategy. In each LOOCV iteration, one experiment is reserved for validation while the remaining 16 runs are used for training. The input features for the residual learner consist of the experimental conditions (initial MG concentration, inoculum amount, incubation time) along with the dynamic-model prediction ${\mathrm{y}}_{\mathrm{dyn},\mathrm{i}}$.

### Feature scaling

To prevent data leakage, the features are scaled to the [0,1] range using a Min–Max scaler, which is fitted only on the training fold in each LOOCV iteration.

### Baseline model calibration

A dynamic response-surface model is first calibrated using the experimental data. This model provides baseline predictions of MG removal (${\mathrm{y}}_{\mathrm{dyn},\mathrm{i}}$) as a function of the operating variables (initial dye concentration, inoculum size, and incubation time).

### Residual learning

The residual (Eq. 1) for each experimental run is computed as the difference between the actual experimental value ${\mathrm{y}}_{\mathrm{actual},\mathrm{i}}$ and the prediction of dynamic model ${\mathrm{y}}_{\mathrm{dyn},\mathrm{i}}$:

1 $${r}_{i}={y}_{actual,i}-{y}_{dyn,i}$$

### Stand-alone machine-learning (ML) regressors

Standalone ML models were first trained to predict MG removal directly from the experimental inputs without using residual corrections. The following regressors were evaluated: Linear models: Ridge, Kernel- and distance-based models: k-Nearest Neighbors (KNN) and Support Vector Regression (SVR)Tree-based models: Decision Tree, Ensemble models: Random Forest and Gradient Boosting, Gradient-boosting libraries: XGBoost, LightGBM and CatBoost, Meta-ensemble: a Voting Regressor combining tuned XGBoost, tuned LightGBM and CatBoost. These models were implemented in Python using scikit-learn, XGBoost, LightGBM, and CatBoost libraries. Their role was twofold: (i) to provide a purely data-driven baseline and (ii) to act as candidate residual learners in the hybrid framework using the feature vector ${\mathrm{x}}_{\mathrm{i}}=(\mathrm{C}{0}_{\mathrm{i}},\mathrm{I}\mathrm{n}\mathrm{o}{\mathrm{c}}_{\mathrm{i}},\mathrm{T}\mathrm{i}\mathrm{m}{\mathrm{e}}_{\mathrm{i}},{\mathrm{y}}_{\mathrm{dyn},\mathrm{i}})$.

### Model hyper parameter tuning

For the linear model (Ridge CV), the regularization parameter ${\upalpha}$ is selected from a logarithmic grid between ${10}^{-3}$and ${10}^{3}$ based on cross-validation. For nonlinear models (KNN, Random Forest, Gradient Boosting), hyper parameters like the number of neighbors (KNN), the number and depth of trees (Random Forest), and the learning rate and depth (Gradient Boosting) are selected based on preliminary trials and established practices.

### Model evaluation

Each model is evaluated on performance metrics including R², RMSE, MAE, MAPE, and sMAPE [31–33] see Eqs. (2)–(6). The LOOCV predictions for each model are used to compute these metrics.

2 $${{\rm{R}}^2} = {\rm{}}1 - {{\mathop \sum \nolimits_{i = 1}^n {{\left( {{{\rm{y}}_i} - {{{\rm{\hat y}}}_i}} \right)}^2}} \over {\mathop \sum \nolimits_{i = 1}^n {{\left( {{{\rm{y}}_i} - {{{\rm{\hat y}}}_i}} \right)}^2}{\rm{}}}}$$

3 $${\rm{RMSE}} = \sqrt {{1 \over n}} \sum\nolimits_{i = 1}^n {{{\left({{{\rm{y}}_i} - {{{\rm{\hat y}}}_i}} \right)}^2}}$$

4 $${\rm{MAE}} = \left( {1/{\rm{n}}} \right)\sum\nolimits_{i = 1}^n {|\left( {{y_i} - {{\hat y}_i}} \right)} $$

5 $${\rm{MAPE}} = \left({100/{\rm{n}}} \right)\sum\nolimits_{i = 1}^n {|\left({{{\rm{y}}_i} - {{{\rm{\hat y}}}_i}} \right)/{{\rm{y}}_i}}$$

6 $${\rm{sMAPE}} = \left({200/{\rm{n}}} \right)\sum\nolimits_{i = 1}^n {{{\left| {{{\rm{y}}_i} - {\rm{}}{{{\rm{\hat y}}}_i}} \right|} \over {\left| {{{\rm{y}}_i}} \right| + \left| {{{{\rm{\hat y}}}_i}} \right|{\rm{}}}}}$$

### Final hybrid model

After evaluating the performance of the individual models, the hybrid model is created by combining the dynamic baseline model with the residuals predicted by the selected machine learning model. The final prediction for each experiment is (Eq. 7):

7 $${y}_{hybrid,i}={y}_{dyn,i}+{\widehat{r}}_{i}$$

The hybrid model is selected based on the lowest LOOCV RMSE and the highest R².

### Confidence intervals

**Fig. 2:**
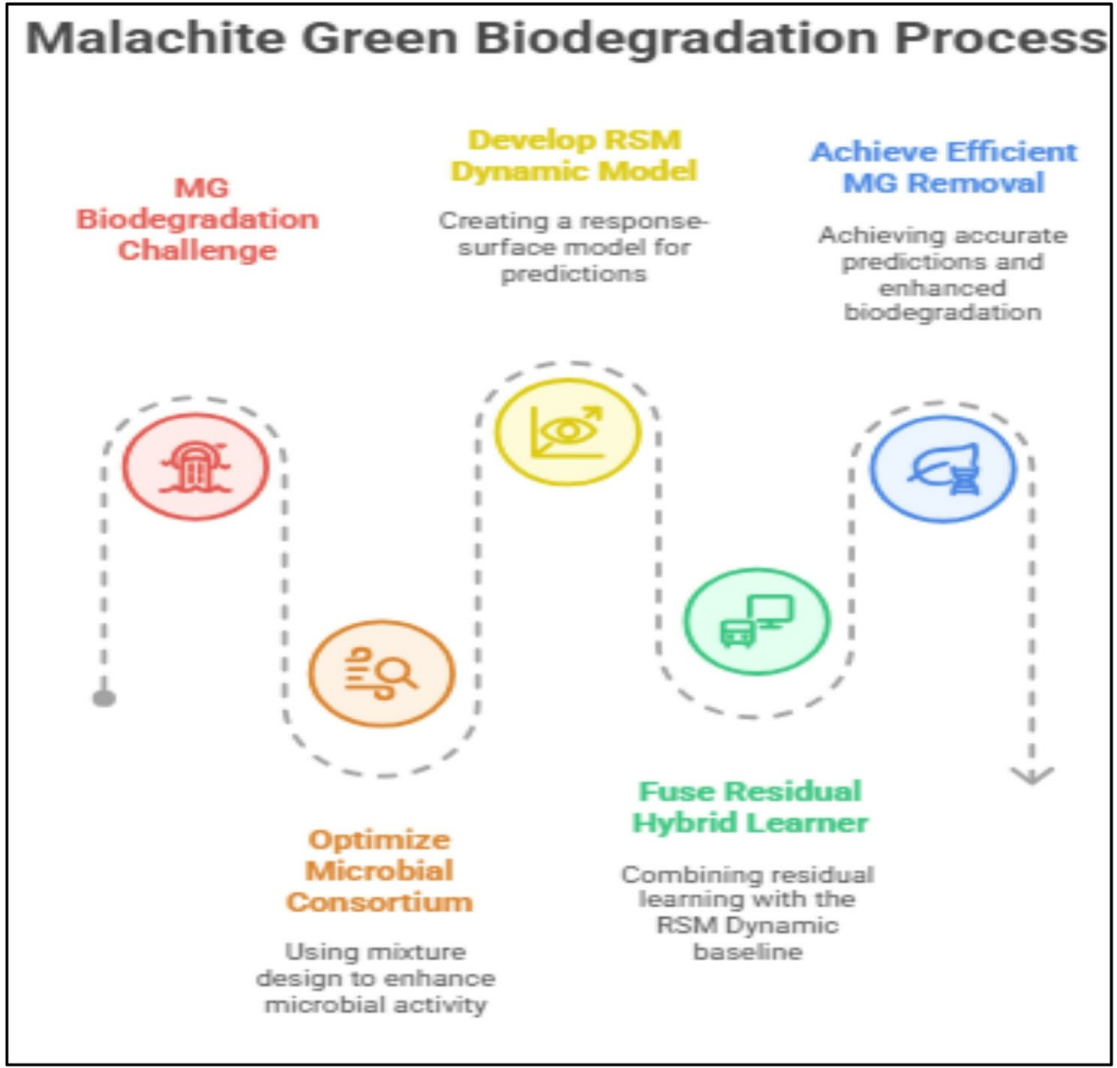
Malachite green biodegradation process: workflow for efficient removal and enhanced biodegradation

95% bootstrap confidence intervals for R² and RMSE are computed using non-parametric resampling of the LOOCV prediction errors (1000 resamples). These intervals provide more robust insight into the performance of model. The final selected model, based on LOOCV performance, is the dynamic RSM model combined with Ridge CV residual learning, which provided the best balance between prediction accuracy and model complexity. This chosen model is well-suited to the small dataset, avoiding overfitting, while also improving predictive performance beyond the mechanistic model. The overall workflow is shown in Fig. 2, and the pseudocode is in Figure S1.

### Extraction and analysis of MG biodegradation products

Several analytical methods were used to examine and validate the biodecolorization and degradation of MG by the formulated microbial consortium. These included the use of UV–vis spectrophotometry to track absorbance changes, Fourier Transform Infrared Spectroscopy (FTIR) to detect changes in functional groups, and Liquid Chromatography-Mass Spectrometry (LC-MS) to detect degradation products and intermediates. The effectiveness of MG biodegradation were confirmed by these complementary methods working together.

### Decolorization analysis via UV–vis spectroscopy analysis

MG dye before and after its decolorization by the formulated consortium under optimized conditions was monitored using a UV-visible spectrophotometer (JASCO V-730 UV-visible/NIR double-beam spectrophotometer, Tokyo, Japan). The MG dye was scanned in the 400–700 nm This spectrophotometric analysis facilitated a quantitative assessment of the MG removal by observing changes in absorbance.

### Analysis of metabolic intermediates after MG dye decolorization through FTIR and LC–MS analysis

After complete decolorization, 100 mL of the degraded sample was centrifuged at 10,000 rpm for 10 min to separate the cell-free supernatant. The supernatant was then extracted with ethyl acetate in a 1:1 ratio, and the extracted solvent was evaporated to dryness, yielding a solid crude extract. This dried residue was subsequently used for further chemical analyses to characterize the biodegradation products of MG.

The samples were analyzed by the FTIR spectrum using a Vertex 70 FTIR spectrometer from Bruker Optik GmbH, Germany, using KBr pellet method in the spectral range of 400 and 4,000 cm^− 1^ with the resolution of 4 cm^− 1^ using 50 scans per sample. The extracts were separated and identified by liquid chromatography coupled to mass spectroscopy using a XEVO TQD triple quadruple instrument, Milford, MA01757 USA, mass spectrometer. The separation was performed on C_18_ column (ACQUITY UPLC - BEH C 18 1.7 μm − 2.1 × 50 mm). The LC flow rate was 0.2 mL/min. The system conditions were mobile phase A- water containing 0.1% formic acid, mobile phase B-methanol containing 0.1% formic acid and column temperature was kept at 30 °C. 10 µL of extract solution was injected. Pure H_2_O was chosen as solvent for analysis. With an ambient pressure electroscopy interface, the LC system was coupled with a Waters mass spectrometer. In positive and negative ionization mode, the electrospray ionization (ESI) source was set.

### Toxicity assessment

The original and the degraded dyes were assayed for their microbial toxicity (1.0 mg/mL) using the well diffusion method. The growth inhibition was in terms of the halo zone against Gram-negative bacteria (*Pseudomonas aeruginosa* ATCC 9027, *Serratia marcescens* 98027, *Escherichia coli* 98082, *Salmonella typhimurium*, *Enterobacter aerogenes* 9805) and Gram-positive bacteria (*Staphylococcus aureus* 91161 and *Bacillus cereus* strain 151007-R3-K09-40–27 F). The data collected were expressed as means and ± standard analysis.

### Molecular differentiation of bacteria strains using PCR based inter simple sequence repeats (ISSR) amplification(PCR-ISSR)

#### Extraction of genomic DNA

A single colony of the bacterial isolate was cultured in a conical flask containing 20 mL of an LB medium by shaking in an orbital shaker at 150 rpm for 18 h. The culture was centrifuged at 13,000 rpm for 5 min at 4 °C. The pellet was subjected to genomic DNA extraction using the (QIAamp DNA Mini Kit, QIAGEN, Germany). The extracted DNA was used as a template for PCR to amplify the DNA fragment flanked by ISSR.

#### PCR-ISSR

Twelve primers, synthesized by Macrogen, Inc. (South Korea), listed in Table S2, amplify DNA with polymorphic and monomorphic bands. ISSR amplifications were performed in 25 µL reactions consisting of 2 µL of 10 pmol primers, 2 µL of DNA template, 12.5 µL of PCR Master Mix 2x concentration (Thermo scientific, USA), and 8.5 µL of deionized H_2_O. PCR condition was done as follow: initial denaturation at 95 °C for 3 min, followed by 35 cycles of 30 s at 94 °C, 30 s at 45 °C, 2 min at 72 °C, and final extension of 10 min at 72 °C using a GeneAmp PCR System 2400 Thermal cycler (Perkin-Elmer Norwalk, Connecticut, USA). The amplification products were resolved by agarose electrophoresis in a 1.5% agarose gel. PCR products were visualized on UV light and photographed using a Gel Documentation System (BIO-RAD 2000).

### Data analysis

Clear and distinct amplification products were scored as ‘1’ for presence and ‘0’ for the absence of bands. The genetic relationships among the examined samples were analyzed using NTSYSpc software, version 2.10e [34]; Exeter Software, Setauket, NY, USA). The binary data matrix derived from the molecular markers was used to compute pairwise genetic similarity coefficients according to the method of Jaccard. The similarity matrix was subsequently subjected to cluster analysis employing the unweighted pair-group method with arithmetic mean (UPGMA) [35] algorithm. A dendrogram was generated to illustrate the genetic relationships among the studied genotypes. The fidelity of clustering was evaluated by calculating the cophenetic correlation coefficient between the similarity and cophenetic matrices.

## Results and discussion

### Decolorization of MG by a bacterial consortium using a D-optimal MD

Numerous studies on microbial consortia with improved degrading capacities have been conducted recently. There is potential for these collaborations to improve the breakdown of dye contaminants in wastewater from textiles [12]. The D-optimal MD was utilized to develop a predictive model illustrating the influence of formulation components on MG removal efficiency (Table 1). This study introduced, for the first time, a bacterial consortium consisting of *A. chroococcum*, *A. salinestris*, *S. maltophilia*, and *S. kyeonggiensis*. The model aids in understanding how these individual species, when combined, contribute to enhanced MG degradation, thereby optimizing the removal process.

### Model fitting

This study confirmed that changes in the MD significantly affect MG decolorization in the consortium compared to monoculture. The actual and predicted MG reduction percentages ranged from 28.53% to 83.51% and from 29.52% to 83.63%, respectively (Table 1). The co-metabolic activity of the individual bacterial strains in the consortium leads to enhanced MG removal, indicating that each strain plays a complementary role, resulting in a synergistic effect that improves decolorization efficiency. The highest decolorization yield of 83.51% was achieved when *A. chroococcum* 6FE and *S. maltophilia* were present in nearly equal proportions of 49.999% and 50.001%, respectively. The microbial synergy and precise formulation for optimizing MG dye biodegradation was illustrated by a linear regression model (Eq. 8).

8 $$\eqalign{ {\rm{MG}}\,{\rm{decolorization}}\left({\rm{\% }} \right){\rm{ = 0}}& {\rm{.68648* X1 + 0}}{\rm{.33026* X2}} \cr & {\rm{ + 0}}{\rm{.67675* X3 + 0}}{\rm{.29521* X4}} \cr & {\rm{ + 0}}{\rm{.010654* X1* X2 + 6}}{\rm{.19E - 03*X1 X3}} \cr & {\rm{ + 6}}{\rm{.12E - 03* X1* X4}} \cr & {\rm{ + 9}}{\rm{.78E - 03* X2 * X3}} \cr & {\rm{ + 9}}{\rm{.51E - 04* X2 * X4}} \cr & {\rm{ + 2}}{\rm{.49E - 03* X3 * X4}} \cr & {\rm{ - 6}}{\rm{.44E - 04 * X1 * X2 * X3}} \cr & {\rm{ - 1}}{\rm{.13E - 03* X1 * X2* X4}} \cr & {\rm{ - 2}}{\rm{.82E - 04* X1* X3 * X4}} \cr & {\rm{ + 1}}{\rm{.73E - 03* X2*X3 * X4}} \cr}$$

where *X1*; *A. chroococcum*, *X2*; *A. salinestris*, *X3*; *S. maltophilia*, and *X4*; *S. kyeonggiensis*.

The analysis of variance (ANOVA) results (Table 2) confirmed the statistical significance of the regression model for MG removal, with a p-value less than 0.0001, indicating a confidence level of 95%. Significant factors showed higher F-values, with the model exhibiting an F-value of 231.19. Among the variables, the linear mixture term had the greatest influence on MG removal (F-value = 734.09), followed by interaction terms such as AB (F-value = 283.68), BC (F-value = 239.52), AC (F-value = 95.89), and AD (F-value = 93.81). The regression model had an excellent fit to the experimental data, with an R² value of 0.998, meaning that 99.8% of the variance in MG removal is explained by the model. These statistics validate the reliability of the model in predicting MG removal efficiency based on the mixture component proportions. This demonstrated a robust relationship between observed and predicted data, so validating the dependability of the model [15]. The models demonstrated strong predictive capability for MG removal, as indicated by a high adjusted R² value of 0.9937. This suggests that the model effectively accounts for variability in MG removal across different operational conditions. The model fits the experimental data well, according to the Lack of Fit test, which produced an F-value of 0.73 and a p-value of 0.4322 in the optimal MD for MG removal. This non-significant outcome confirms the suitability of model and implies that the optimization outcomes are accurate and suitable for practical use in dye removal. Additionally, the quadratic model’s non-significant lack of fit value (> 0.05) indicated that it is statistically significant for the response and can thus be utilized for more investigation [36, 37]. Together, these statistics validate the models as robust and accurate tools for forecasting MG removal efficiency.

### Interpretation of contour and surface plots

**Fig. 3:**
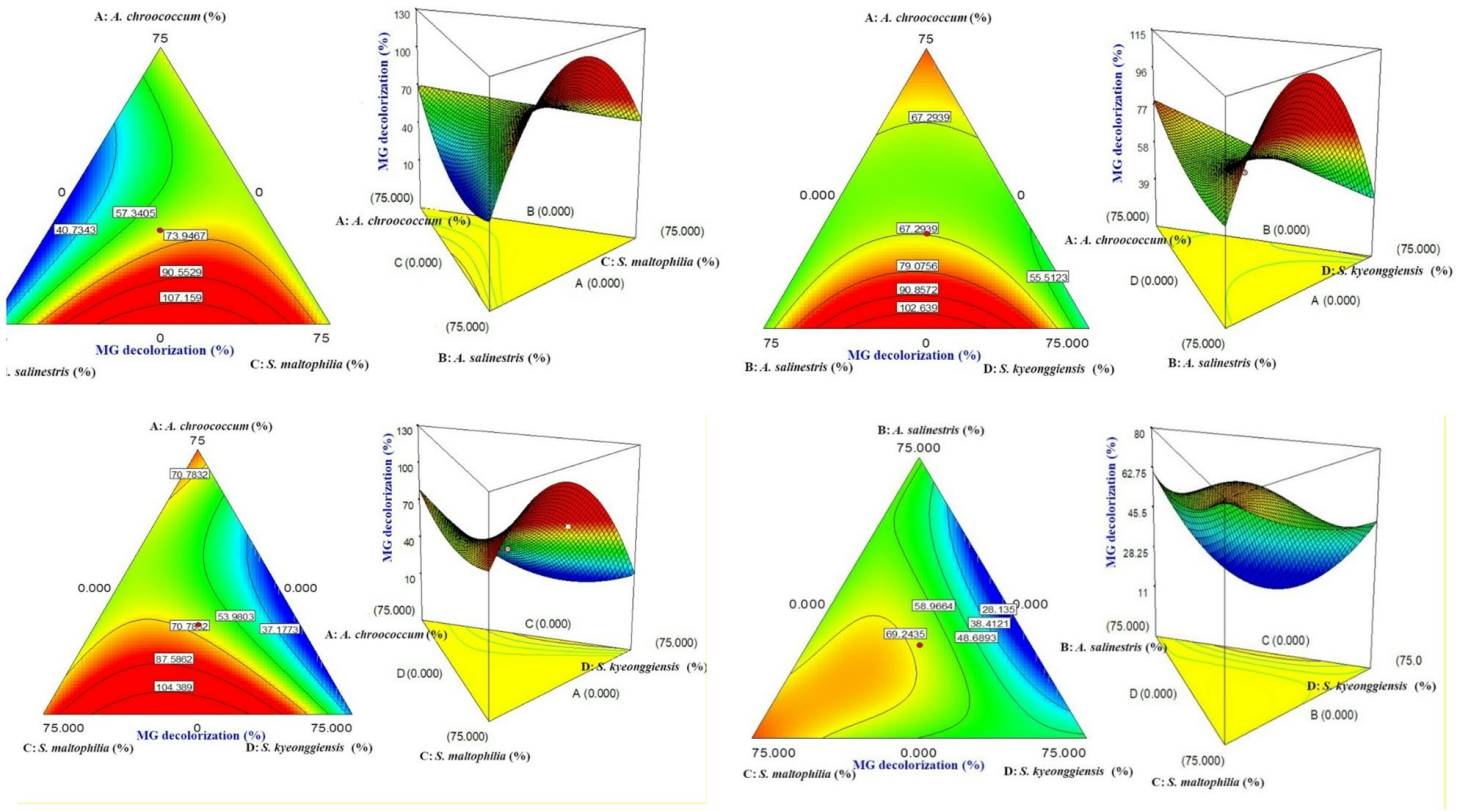
Mixture contour and response surface plots showing the interactive effects among the inoculum variables — *A. chroococcum*, *A. salinestris*, *S. maltophilia*, and *S. kyeonggiensis*— on malachite green (MG) removal efficiency

The interaction between the independent variables influencing MG removal is visualized using D-optimal surface and contour plots. Figure 3 demonstrates how the proportions of *A. chroococcum*,* A. salinestris*,* S. maltophilia*, and *S. kyeonggiensis* interact to affect MG removal efficiency. On these plots, response values range from low (indicated by dark blue areas representing 28.53% removal) to high (indicated by dark red areas representing 83.51% removal). These visualizations help illustrate the synergistic effects and optimal combinations of the microbial consortium for effective dye degradation. Such contour plots are effective tools for interpreting complex variable interactions in MD and for identifying conditions that maximize the desired response.

Elliptical contours typically suggest an ideal interaction between the independent variables, reflecting the harmonious effects of the component mixtures on the response [38]. This visualization helps pinpoint the best combination of components to achieve the desired outcomes. The highest decolorization of MG was observed with the bacterial strain *A. salinestris* (X2) in combination with *S. maltophilia* (X3), as indicated by the optimal red zone in the graphical results (Fig. 3). This increased decolorization efficiency for binary mixtures is attributed to synergistic decolorization behaviors, reinforcing the role of microbial partnerships in enhancing dye degradation. Notably, *S. maltophilia* is well-documented for its effective dye decolorization capabilities, including MG [13, 39]. Figure 3 also presents 3-D response surface graphs illustrating the reduction of MG as a function of the mixture components in the bacterial consortium. These surface plots capture both the individual and combined effects of the four strains on MG decolorization, confirming previous findings [40]. This approach streamlines optimization by visually representing the interactions among the mixture components. Also, Table S3 shows the regression coefficients with 95% confidence intervals for MD Model.

### Interpretation of residual graph

Residuals in a fitted model represent the differences between observed responses and the predicted values from the regression equation. Analyzing residual plots is essential for assessing model quality, including assumptions about independence, normality, and the constant variance of residuals [41]. A normal probability plot (Figure S2a) indicated that the residuals from the MG removal data closely align along a straight line, with an even distribution on both sides, suggesting that the residuals follow a normal distribution. The residual values ranged from approximately − 1.69 to + 1.58, supporting the data fit the assumptions of normality, thereby enhancing confidence in the regression analysis [42]. The residual vs. predicted values plot (Figure S2b) displays residuals scattered randomly above and below the zero line, indicating no serial correlation in the data. The studentized residuals vs. run number plot (Figure S2c) shows residuals dispersed randomly within ± 3.00, suggesting a good spread around the mean efficiency of model. Figure S2d compares predicted and actual MG removal values, demonstrating an even distribution and strong agreement. The optimal lambda (λ) of Box-Cox plot (Figure S2e) was 1.23 and values within the 95% confidence interval (− 0.470 to 3.33) had no transformation.

This study represents the first application of *A. chroococcum*,* A. salinestris*, and *S. kyeonggiensis* for the removal of MG dye, with no prior reports of their consortia. In contrast, *S. maltophilia* has been documented in limited studies related to dye degradation including MG [43]. In microbial consortia, different bacterial strains contribute to degradation by targeting distinct sites on the aromatic rings of dye or by metabolizing intermediates produced by other strains, which facilitates further breakdown [44, 45]. Our consortium demonstrated greater effectiveness in MG decolorization compared to individual pure cultures due to their metabolic diversity and enzymatic pathways involved [46]. Enzymes which might be involved in the bioremediation of MG were laccase [47], Mn-Peroxidase [48], dichlorophenolindophenol (DCIP) reductase [49], malachite green reductase, NADH-DCIP reductase, and tyrosinase [50]. These enzymes aid in biodegradation by catalyzing the conversion of MG dye into less hazardous or colorless metabolites via oxidation or reduction pathways.

### Optimization of MG decolorization via BBD

An optimization study on the decolorization of MG using a bacterial consortium of *A. chroococcum* and *S. maltophilia* was conducted employing RSM. The highest MG removal rate of 96.56% was achieved in run 10, with a 100 mg/L MG concentration, a 7% inoculum size, and a 72-h incubation period (Table 3). The relationship between these variables and MG removal was modeled using regression equation expressed in coded values, as shown in Eq. (9).

9 $$\eqalign{ {\rm{MG}}\,{\rm{removal}}\left({\rm{\% }} \right)& {\rm{ = 111}}{\rm{.60228 - 0}}{\rm{.037247*A}} \cr & {\rm{ - 3}}{\rm{.82285*B - 0}}{\rm{.12161*C}} \cr & {\rm{ + 0}}{\rm{.01457*A*B + 5}}{\rm{.55E04*A*C}} \cr & {\rm{ + 6}}{\rm{.10E03*B*C - 2}}{\rm{.33E - 04*}}{{\rm{A}}^{\rm{2}}} \cr & {\rm{ - 0}}{\rm{.04656*}}{{\rm{B}}^{\rm{2}}}{\rm{ + 2}}{\rm{.23E - 03*}}{{\rm{C}}^{\rm{2}}} \cr}$$

where A: dye concentration, B; inoculum size, and C; incubation time.

Equation (9) demonstrates the influence of interactive model terms on MG removal by the bacterial consortium. Within this equation, a positive coefficient indicates a synergistic effect between variables, meaning they work together to enhance MG removal. Conversely, a negative coefficient signifies an antagonistic effect, where the interaction between factors reduces the overall removal efficiency.

The adequacy of the suggested quadratic model for MG removal was evaluated using ANOVA (Table 4). The model exhibited a high F-value of 66.27 and a p-value of less than 0.05, indicating statistical significance. The high R² (0.9884), along with the adjusted R² (0.9735) and predicted R² (0.9425) values confirms the accuracy of the model. Additionally, a low CV (5.55%) indicates good precision and reliability of the experimental data. Statistically significant terms (*p* < 0.05) included the linear effects of variables A, B, and C, the interaction term AB, and the quadratic term A², demonstrating that there is sufficient experimental data to explain the effects of these process parameters on MG decolorization. When the p-value was 0.7552 > 0.05, the lack of fit value F-0.41 in the current BBD model showed that it was not significant in relation to pure error. This non-significant result supports the validity of our findings by indicating that the model appropriately describes the data without major deviations [51, 52]. Table S4 shows regression coefficients with 95% confidence intervals.

**Table 4:** ANOVA for quadratic response surface model developed by BBD for MG dye removal

Source	Sum of Squares	df	Mean Square	F-Value	*P*-value	
Model	9954.82	9	1106.09	66.27	< 0.0001	significant
A-A	6551.92	1	6551.92	392.55	< 0.0001	
B-B	195.15	1	195.15	11.69	0.0111	
C-C	586.72	1	586.72	35.15	0.0006	
AB	687.83	1	687.83	41.21	0.0004	
AC	63.8	1	63.8	3.82	0.0915	
BC	0.77	1	0.77	0.046	0.8358	
A^^2^	1858.77	1	1858.77	111.37	< 0.0001	
B^^2^	0.74	1	0.74	0.044	0.8393	
C^^2^	6.93	1	6.93	0.42	0.5398	
Residual	116.83	7	16.69			
Lack of Fit	27.47	3	9.16	0.41	0.7552	non-significant
Pure Error	89.37	4	22.34			
Cor Total	10071.65	16				

Std. Dev.= 4.09; Mean = 73.62; C.V. %= 5.55; PRESS = 579.12; R^2^ = 0.9884; Adj R^2^ = 0.9735; Pred R^2^ = 0.9425; Adeq Precision = 26.637

A: Initial dye concentration, B: Amount of consortia inoculum, C: Incubation time (h)

**Fig. 4:**
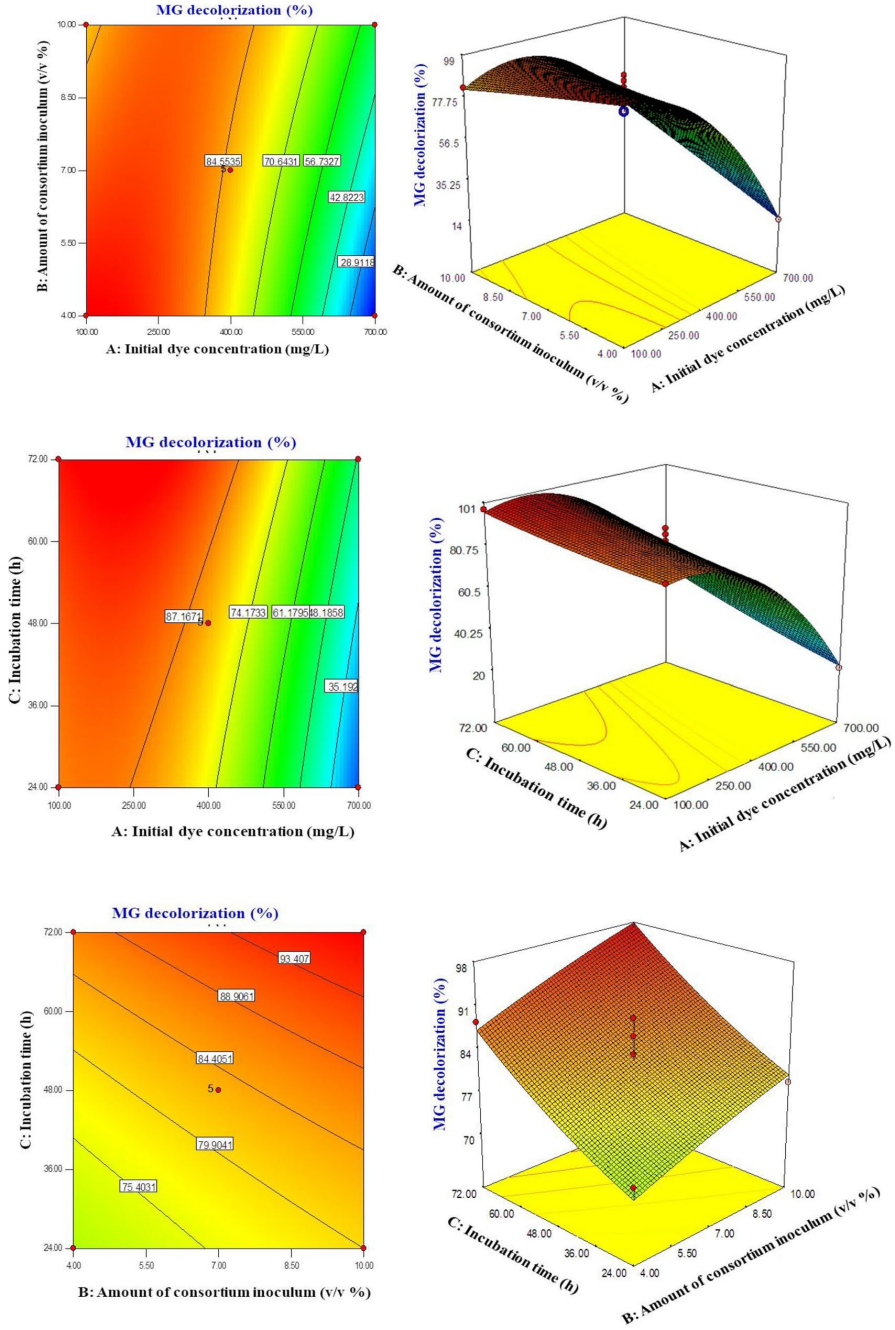
2D contour and 3D surface and plots of malachite green (MG) decolorization via the bacterial consortium (*A. chroococcum* and *S. maltophilia*), showing the interaction effects among the independent variables

The interaction between factors affecting MG decolorization was explored using 2D and 3D response surface plots (Fig. 4). The removal efficiency decreased as MG concentration increased from 100 to 700 mg/L, likely due to the saturation of enzyme binding sites at higher dye levels. Incubation time significantly influenced MG removal, achieving up to 97.81% decolorization. When considering both consortium dosage and incubation time, MG removal efficiency improved with larger inoculum sizes and longer incubation periods, reaching optimal discoloration at a 7% consortium dosage and 72 h of incubation. These findings emphasize the necessity of optimizing bacterial consortium concentration and incubation duration for effective MG decolorization.

The normal probability plot of residuals for MG removal (Figure S3a) shows data points symmetrically arranged around the central line. This confirms the appropriateness of the BBD used for modeling MG removal. Additionally, the plot of experimental versus predicted MG decolorization efficiencies (Figure S3b) shows points clustered near the diagonal line with minimal deviation, indicating a good fit of the quadratic model to the experimental data.

For bacteria to be effective in industrial applications, they must efficiently degrade or decolorize target dyes without inhibition at relevant concentrations. However, increasing dye concentrations often result in decreased decolorization rates. This decline is typically due to the toxicity of high dye levels, which inhibits bacterial metabolic activities [53, 54]. Also, the higher concentrations (e.g., 700 mg/L) obstruct the enzymes active sites thereby reducing microbial degradation efficiency [55]. Current data indicate that optimal decolorization occurs at lower dye concentrations. Therefore, optimizing dye concentration is crucial for maximizing decolorization efficiency in practical applications.

The cell-to-dye ratio is a critical factor influencing the efficiency of MG bioremediation. At low inoculum sizes (e.g., 4%), decolorization decreases, likely due to slower bacterial growth and reduced enzyme production, which limits degradation as dye molecules saturate the active sites of enzyme. In contrast, increasing the inoculum size promotes bacterial proliferation and enzyme activity, significantly enhancing decolorization efficiency [56]. In this study, the highest MG removal was observed at a 7% inoculum size (for 100 mg/L dye), indicating optimal conditions for bacterial growth and activity. However, beyond this optimal inoculum size, efficiency declined, likely due to nutrient depletion and excessive biomass, which reduced microbial metabolic activity and enzyme production [57]. This highlights the importance of optimizing inoculum size to maximize dye degradation without adversely affecting bacterial performance. Incubation time is a vital aspect influencing the efficiency of MG decolorization. The study revealed that a longer incubation period, specifically 72 h, resulted in the highest MG removal, thus provide a sufficient time for microbial growth and enzymatic activity.

### Comparison between experimental designs of MD and BBD

**Fig. 5:**
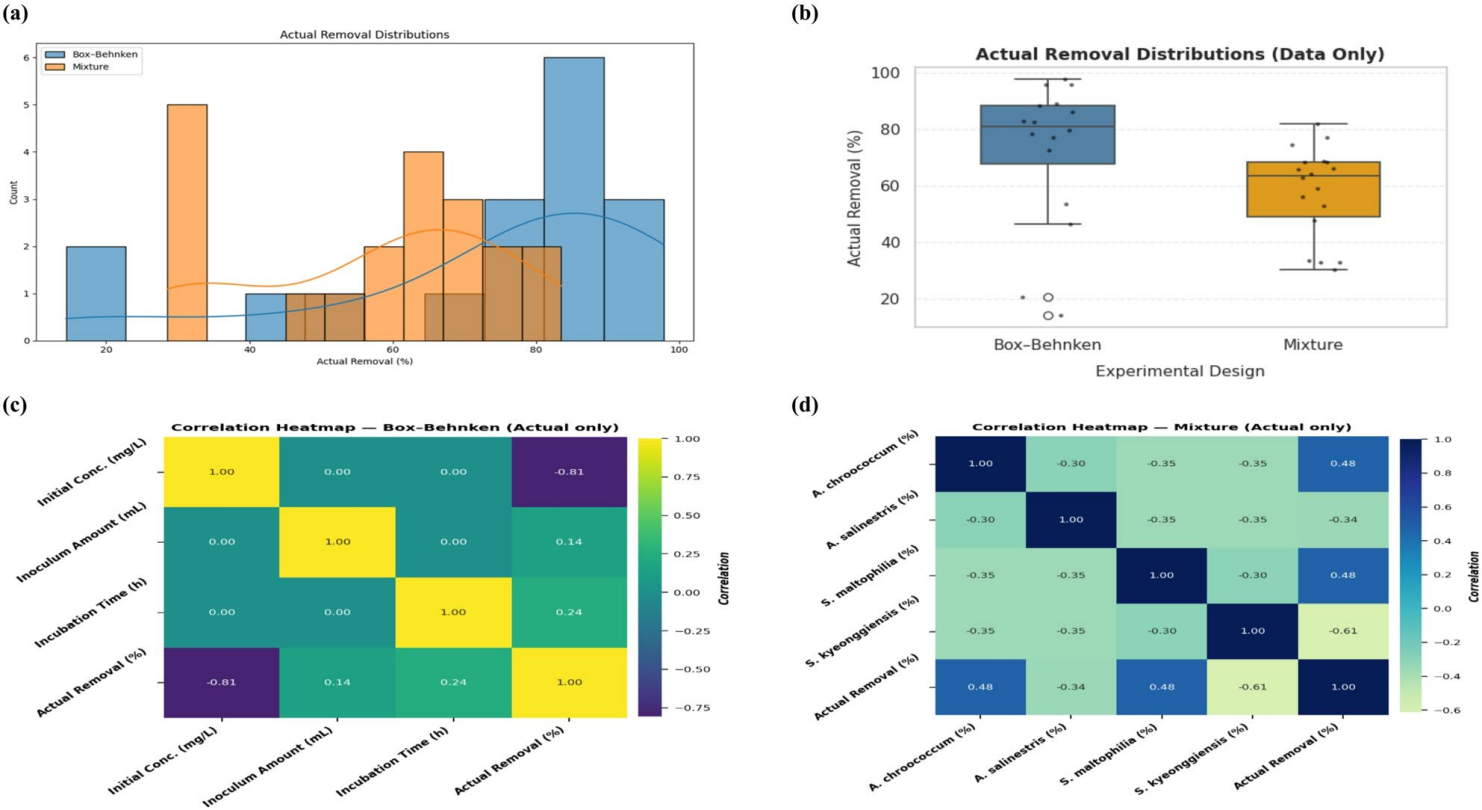
Comparative analysis of actual MG removal performance across experimental designs: (**a**) boxplots comparing actual removal efficiencies between the Box–Behnken and Mixture designs, with Box–Behnken exhibiting higher and more consistent performance; (**b**) histograms with kernel density estimates of actual removal, showing a stable unimodal profile for Box–Behnken and a bimodal distribution for the Mixture design; (**c**) correlation heat map for the Box–Behnken design (actual values only), highlighting a strong negative correlation of initial dye concentration with removal efficiency; (**d**) correlation heat map for the Mixture design (actual values only), showing positive associations of *A. chroococcum* and *S. maltophilia* with removal efficiency and a negative association of *S. kyeonggiensis*

The comparison between experimental designs is illustrated in Fig. 5a. The BBD yielded consistently higher MG removal efficiencies, with median values above 80% and a compact distribution. In contrast, the MD exhibited broader variability and lower central tendency, reflecting heterogeneous microbial interactions. The distribution of actual MG dye removal values is presented in Fig. 5b. Box–Behnken data cluster around 80–95%, indicating stable performance, whereas the MD shows a bimodal distribution with groups around 30–40% and 60–70%. This highlights variability in microbial mixture outcomes.

Correlation analysis for the BBD is shown in Fig. 5c. A strong negative correlation (*r* = − 0.81) was found between initial MG dye concentration and removal efficiency, confirming that higher MG concentration suppress treatment performance. Incubation time correlated weakly (*r* = 0.24), while inoculum amount had minimal effect. As shown in Fig. 5d, microbial composition strongly influenced MG dye removal efficiency. *A. chroococcum* and *S. maltophilia* displayed positive correlations (r ≈ + 0.48), whereas *S. kyeonggiensis* was negatively correlated (*r* = − 0.61). These results emphasize the role of microbial interactions in MG treatment performance.

The scatter matrix in Figure S4a visualizes pairwise relationships among variables in the BBD. A clear negative trend is observed between initial MG concentration and removal efficiency, while incubation time and inoculum amount show weaker associations. Figure S4b expresses the scatter matrix for the MD, showing clustered patterns linked to discrete microbial strain proportions. Runs with dominant single strains (100%) formed distinct groups, whereas mixed compositions yielded intermediate efficiencies, reflecting nonlinear interactions.

### Machine learning and hybrid residual modeling framework

In addition to the two experimental datasets summarized in Table 1 (MD) and Table 3 (BBD), an RSM-based dynamic response-surface model was constructed using Design-Expert^®^ v7.0 to simulate the MG decolorization process over the experimental design space. The predictions of this RSM baseline played a dual role: first, as a structured, design-based benchmark against which the experimental results could be evaluated, and second, as the baseline component in the hybrid residual ML models (experimental MG removal minus RSM prediction). This integration of experimental observations with a response-surface baseline follows established hybrid-modeling practices reported in the literature—such as hybrid residual correction schemes in chemical engineering and physics-enhanced residual learning in dynamical systems [58].

**Fig. 6:**
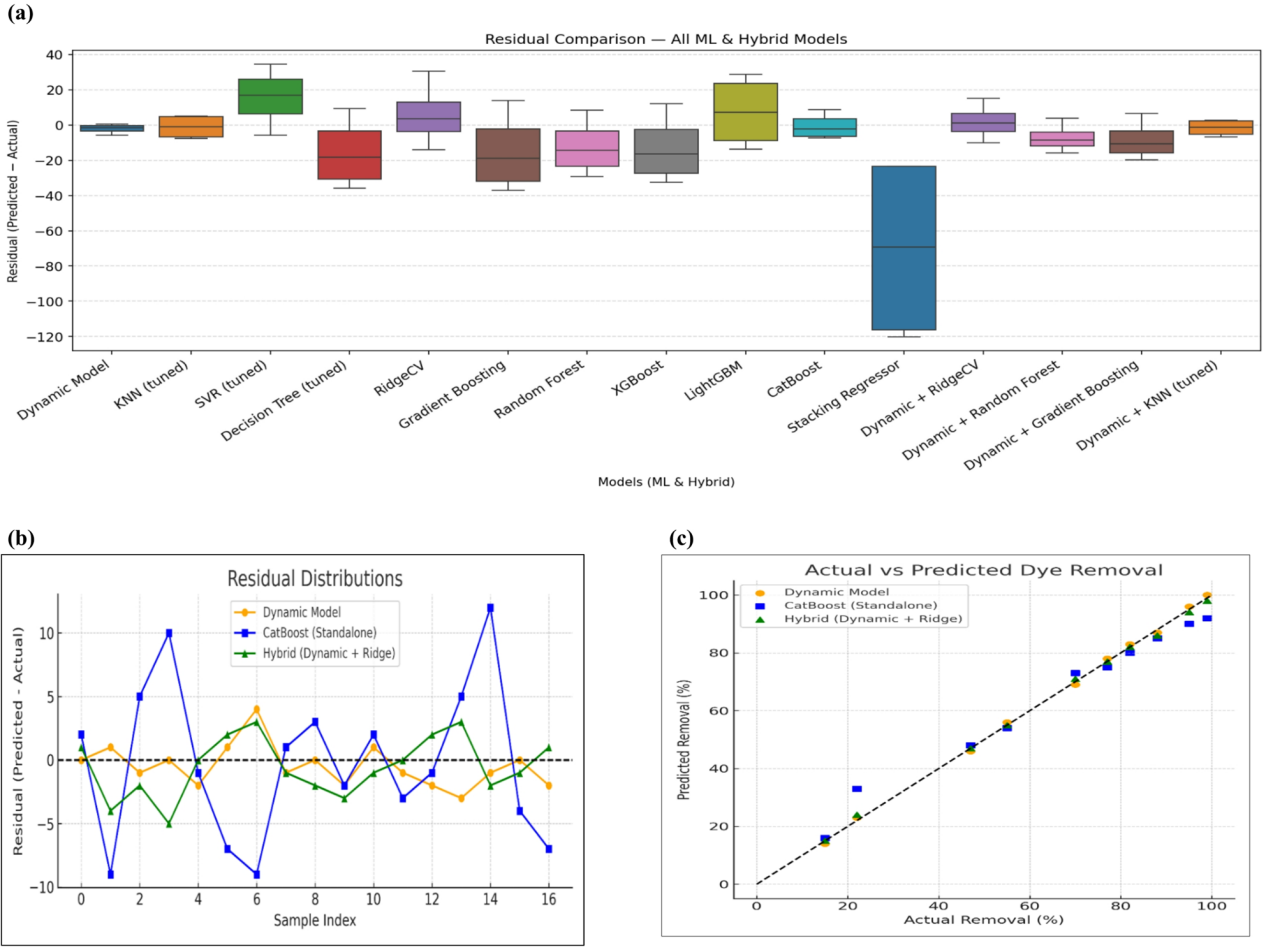
Residual distributions (Predicted − Actual) for the Dynamic RSM baseline, standalone machine-learning regressors, and hybrid Dynamic + machine learning (ML) models for MG decolorization. (**a**), Actual vs. Predicted MG Decolorization for Dynamic, Ml, and Hybrid RidgeCV models (**b**), Residual distributions for Dynamic, CatBoost, and Hybrid Ridge CV models (**c**)

The predictive performance of the proposed models was assessed by comparing three families of predictors: (i) the RSM-based dynamic response-surface baseline (“Dynamic Model”), (ii) standalone machine-learning techniques (MLTs), and (iii) residual hybrid learning approaches. The results in Table 5a indicate that the Dynamic Model achieved the highest accuracy (R² = 0.968, RMSE = 3.26, MAE = 2.43, MAPE = 3.38), outperforming all standalone MLTs. KNN and CatBoost performed relatively well (R² ≈ 0.887, RMSE ≈ 6.1) but still lagged the structured RSM baseline. In contrast, ensemble methods such as Random Forest, Gradient Boosting, and LightGBM produced negative R² values, indicating poor predictive capability under the present small-sample constraints. SVR exhibited very weak generalization (R² = − 0.426, RMSE ≈ 21.7). Regularized linear models, such as Ridge, Lasso, and ElasticNet, also showed poor standalone performance when fitted directly to the raw MG-removal data (Table 5a), reflecting the strongly nonlinear and interaction-rich nature of the underlying process. As shown in Figure S5a, the Dynamic Model closely follows the actual trend across all samples, whereas most ML regressors deviate substantially. Ensemble methods and SVR show wide mismatches between predicted and actual values, while CatBoost and KNN track the data more closely but still diverge at several points. The Dynamic Model also exhibits minimal residuals centered near zero (Fig. 6a), confirming its accuracy and stability.

**Table 5:** Performance comparison of the empirical *RSM-based dynamic baseline* with (a) standalone machine-learning regressors evaluated using an 80/20 train–test split, (b) hybrid residual learners benchmarked against this RSM-based dynamic baseline using LOOCV (*N* = 17), and (c) the best-performing models across both evaluation schemes. For the LOOCV-based hybrids in panel (b), 95% bootstrap confidence intervals (CI95%) are reported for R² and RMSE. Confidence intervals were not computed for the standalone models in panel (a) because the test subset contained only four samples, making CI estimation unstable and uninformative

Model	*R*²	RMSE	MAE	MAPE	SMAPE	R2 (95% CI)	RMSE (95% CI)
**(a)**							
Dynamic Model (RSM baseline)	0.9678	3.2641	2.4325	3.3759	3.4601	-	-
KNN (tuned)	0.8871	6.1103	6.0025	9.2771	9.4020	-	-
CatBoost	0.8864	6.1283	5.6132	9.3479	9.3723	-	-
RidgeCV	0.0993	17.2580	13.1001	17.2978	15.9052		
SVR (tuned)	-0.4257	21.7126	18.5700	34.2244	27.1344	-	-
Decision Tree (tuned)	-0.7448	24.0196	20.9112	37.7441	53.1948	-	-
Gradient Boosting	-0.7967	24.3742	20.7103	37.8802	54.3134	-	-
Random Forest	-0.1873	19.8142	17.2375	31.0115	40.5646	-	-
XGBoost	0.0802	17.4402	16.4336	26.9412	30.7696	-	-
LightGBM	-0.1624	19.6053	17.8775	31.5801	26.6855	-	-
Stacking Regressor	-20.7304	84.7677	70.5238	131.6604	115.9013	-	-
**(b)**							
Dynamic Model (RSM baseline)	0.9884	2.6208	2.0318	3.0127	2.9995	0.9220–0.994	1.6024–3.5098
Dynamic (RSM baseline) + RidgeCV	0.9869	2.7850	2.1593	3.2030	3.1881	0.9911–0.9959	1.7027–3.7295
Dynamic (RSM baseline) + RandomForest	0.9818	3.2807	2.4986	3.6562	3.6445	0.8796–0.9948	1.9655–4.3855
Dynamic (RSM baseline) + GradientBoosting	0.9810	3.3558	2.5369	3.5802	3.5744	0.8829–0.9952	1.9016–4.3823
Dynamic (RSM baseline) + KNN	0.9746	3.8822	3.3044	4.8800	4.7805	0.8341–0.9911	2.5912–5.0607
**(c)**							
Dynamic Model (RSM baseline)	0.9884	2.6208	2.0318	3.0127	2.9995	0.9220–0.994	1.6024–3.5098
Best Standalone ML (CatBoost)	0.8864	6.1283	5.6132	9.3479	9.3723	-	-
Best Hybrid (Dynamic (RSM baseline) + Ridge CV)	0.9869	2.7850	2.1593	3.2030	3.1881	0.9911–0.9959	1.7027–3.7295

Note: Among the standalone ML models in panel (a), tuned KNN achieved the highest R² and lowest RMSE, with CatBoost showing very similar performance; however, given the small test set (*n* = 4), these differences should be interpreted with caution

R²: coefficient of determination; RMSE: root mean square error; MAE: mean absolute error; MAPE: mean absolute percentage error; SMAPE: symmetric mean absolute percentage error

Residual hybrid learning improved performance relative to standalone ML, as shown in Figure S5b**.** The RSM-based Dynamic Model again achieved the best accuracy (R² = 0.988, RMSE = 2.62). Linear residual correction using RidgeCV closely matched the baseline (R² = 0.987, RMSE = 2.78; Table 5b), demonstrating the efficiency of simple linear hybrids. Interestingly, although Ridge regression was not competitive as a standalone predictor, it became the most reliable residual learner once the RSM baseline had captured the dominant nonlinear trends; in this setting, the remaining error structure is small in magnitude and approximately linear, making a low-variance linear corrector particularly effective. Ensemble hybrids such as Dynamic + Random Forest and Dynamic + Gradient Boosting exhibited improved performance relative to their standalone counterparts but remained weaker than the baseline (R² ≈ 0.981, RMSE ≈ 3.3). Dynamic + KNN was the least successful hybrid (R² = 0.975, RMSE = 3.88). Residuals for the RidgeCV hybrid and the RSM-based dynamic model remain small and centered near zero (Figure S6), indicating high predictive stability. Notably, the 95% confidence interval for both R² (0.9911–0.9959) and RMSE (1.70–3.73) was narrower than that of all other assessed models, confirming the superior precision and lower uncertainty of the hybrid RidgeCV predictor compared to alternative ML regressors. As shown in Figure S6, the Dynamic RSM baseline and all hybrid Dynamic + ML models (Dynamic + RidgeCV, Gradient Boosting, Random Forest, XGBoost, CatBoost, and KNN) are compared in terms of R² and RMSE, along with 95% confidence intervals for each model.

Residual analyses (Fig. 6a–b) show that both the RSM-based Dynamic Model and the Dynamic + RidgeCV hybrid produce small, nearly zero-centred errors, with the hybrid displaying only a slightly broader spread than the baseline, whereas CatBoost exhibits larger and more dispersed residuals. Consistently, the actual-versus-predicted plot (Fig. 6c) confirms that Dynamic and Dynamic + RidgeCV predictions lie very close to the 1:1 line, while CatBoost deviates more noticeably, particularly at the extremes of MG removal. According to Table 5c, the Dynamic Model outperformed both the best standalone ML model (CatBoost, R² = 0.886, RMSE = 6.13) and the best hybrid model (Dynamic + RidgeCV, R² = 0.987, RMSE = 2.78), achieving the highest overall accuracy (R² = 0.988, RMSE = 2.62). Because the dataset comprises only 17 experiments, we additionally relied on leave-one-out cross-validation (LOOCV) to obtain a statistically efficient estimate of out-of-sample performance. Under LOOCV, the R² of Dynamic Model increased from 0.968 (RMSE = 3.26% MG removal) under the exploratory 80/20 split to 0.988 (RMSE = 2.62), demonstrating that the RSM baseline is highly stable and that LOOCV provides a more reliable benchmark under small-sample constraints.

Overall, these results emphasize the advantage of using a structured, RSM-based dynamic response-surface baseline in data-scarce situations. Standalone ML models, particularly tree-based and boosting methods, exhibited high variance and limited generalization, whereas residual hybrid learning provided a practical compromise: ensemble hybrids outperformed standalone ML but did not surpass the baseline, whereas linear hybrids approached baseline accuracy with balanced error distributions. This behavior aligns with hybrid-modeling findings from other domains, where structured baselines or mechanistic components are combined with residual ML corrections—for example, in spacecraft power-system prediction and in hybrid SEIR–machine-learning frameworks for COVID-19 forecasting [59, 60]. To our knowledge, no previous study has employed a residual hybrid machine-learning framework that integrates a structured baseline model for MG dye removal, highlighting the novelty of the present work.

The hybrid residual-learning strategy adopted in this work, where a mechanistic Dynamic RSM baseline is corrected by ML regressors, is consistent with recent developments in hybrid modelling. **Schweidtmann et al.** [61] reviewed how combining first-principles and data-driven models systematically improves predictive accuracy and reduces experimental effort in complex process systems. Likewise, hybrid physics–ML frameworks have been successfully applied to non-linear environmental and geo-industrial problems, for example in rate-of-penetration prediction [62]. Our results for MG decolorization follow the same trend, with the hybrid Dynamic + ML models clearly outperforming the standalone data-driven models and the RSM baseline.

From an application perspective, the proposed bacterial consortium is compatible with current trends in using microbial consortia for robust bioremediation of industrial pollutants. Recent work has shown that tailored bacterial consortia can efficiently remove complex mixtures of contaminants and are suitable for deployment in full-scale systems [63]. In our case, the observed synergy in MG removal suggests that scale-up to pilot and industrial reactors is feasible, provided that operational variables (e.g. residence time, nutrient supply, aeration) are tuned appropriately. The hybrid model can then serve as a soft sensor or decision-support tool to optimize operating conditions in real time.

At the same time, this work illustrates typical limitations of small-sample ML in environmental systems. The relatively limited number of experimental runs constrains the diversity of operating conditions represented in the training set and may restrict extrapolation beyond the calibrated design space. This is a common challenge in data-driven process and environmental modelling, where experiments are expensive and time-consuming. Future work should therefore extend the dataset and explore regularization, uncertainty quantification, and adaptive or transfer-learning strategies to improve robustness and general ability of the hybrid models.

### Proposed pathway of MG decolorization

**Fig. 7:**
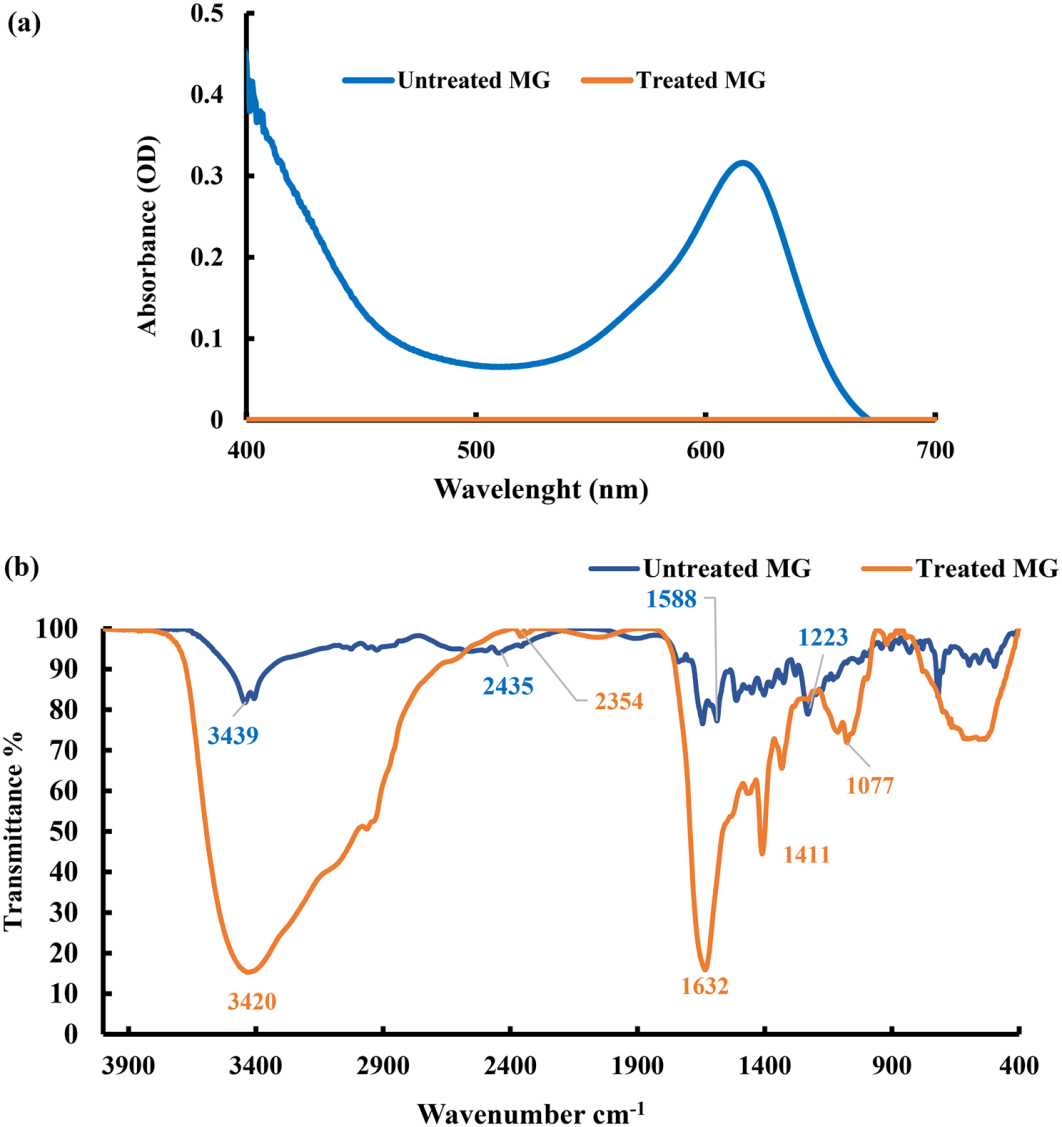
UV–Vis (**a**) and FTIR (**b**) spectra of MG dye before and after treatment with the formulated bacterial consortium

The UV-Vis spectroscopy showed a sharp decrease in the characteristic absorption peak of MG at 618 nm after decolorization indicated that the bacterial consortium effectively cleaves the chromophoric bonds responsible for the color of dye (Fig. 7a). The disappearance of absorbance in the UV region suggests a breakdown of MG. These spectral changes imply that the primary chromophores, which give MG its color, were attacked and biotransformed [64] by the bacterial consortium. This transformation was verified by detection of metabolites using FTIR and LC-MS, confirming molecular modifications consistent with dye degradation. Overall, this showed that the bacterial consortium not only adsorbed MG but also enzymatically degraded it through molecular cleavage, thereby detoxifying the dye and contributing to environmental remediation.

FTIR spectroscopy is commonly used to analyze MG dye degradation. Following biodegradation by a bacterial consortium, the FTIR spectra of MG and its byproducts are depicted in Fig. 7b. The untreated MG spectrum exhibited characteristic absorption bands at 3439, corresponding to aromatic C-N stretching. Additionally, peaks at 2435, 1588, and 1223 cm^− 1^ were corresponded to C = C stretching of benzene rings, C-H asymmetric stretching, and NH asymmetric stretching vibrations. These peaks were slightly shifted to lower values at 3420, 2354, 1411 and 1077 cm^− 1^, respectively. This shift in peak values could be attributed to the biodegradation of MG and the formation of new MG derivatives. This suggests that the bacterial attacked the dye structure, breaking down its key functional groups and transforming MG into different metabolites. The formation of new derivatives is consistent with partial degradation or biotransformation pathways rather than simple adsorption. Overall, the FTIR spectral changes provide molecular-level evidence that the bacterial consortium actively decomposes MG, leading to structural changes in the dye molecules which likely reduce their toxicity and color, ultimately contributing to environmental detoxification.

**Fig. 8:**
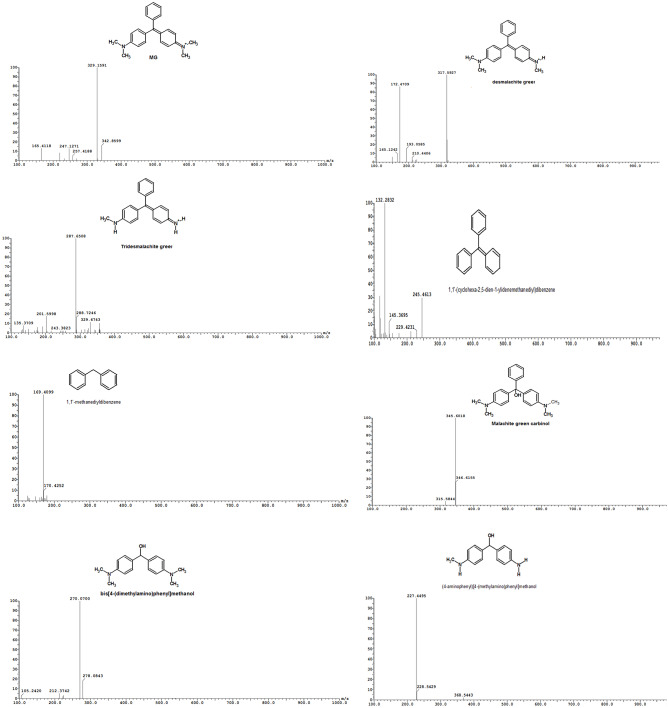

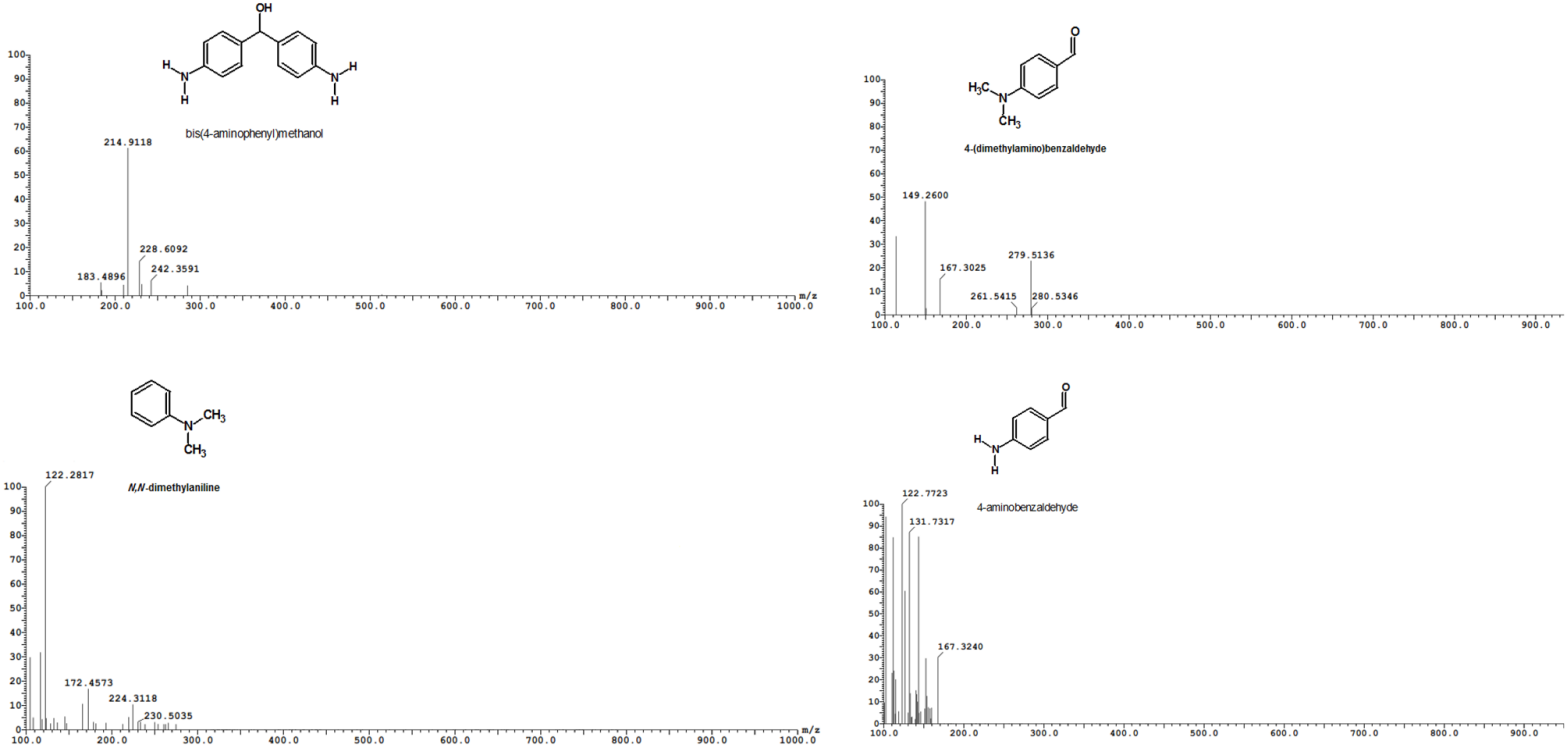
Mass spectra of MG intermediates degradation determined by LC–MS

**Fig. 9:**
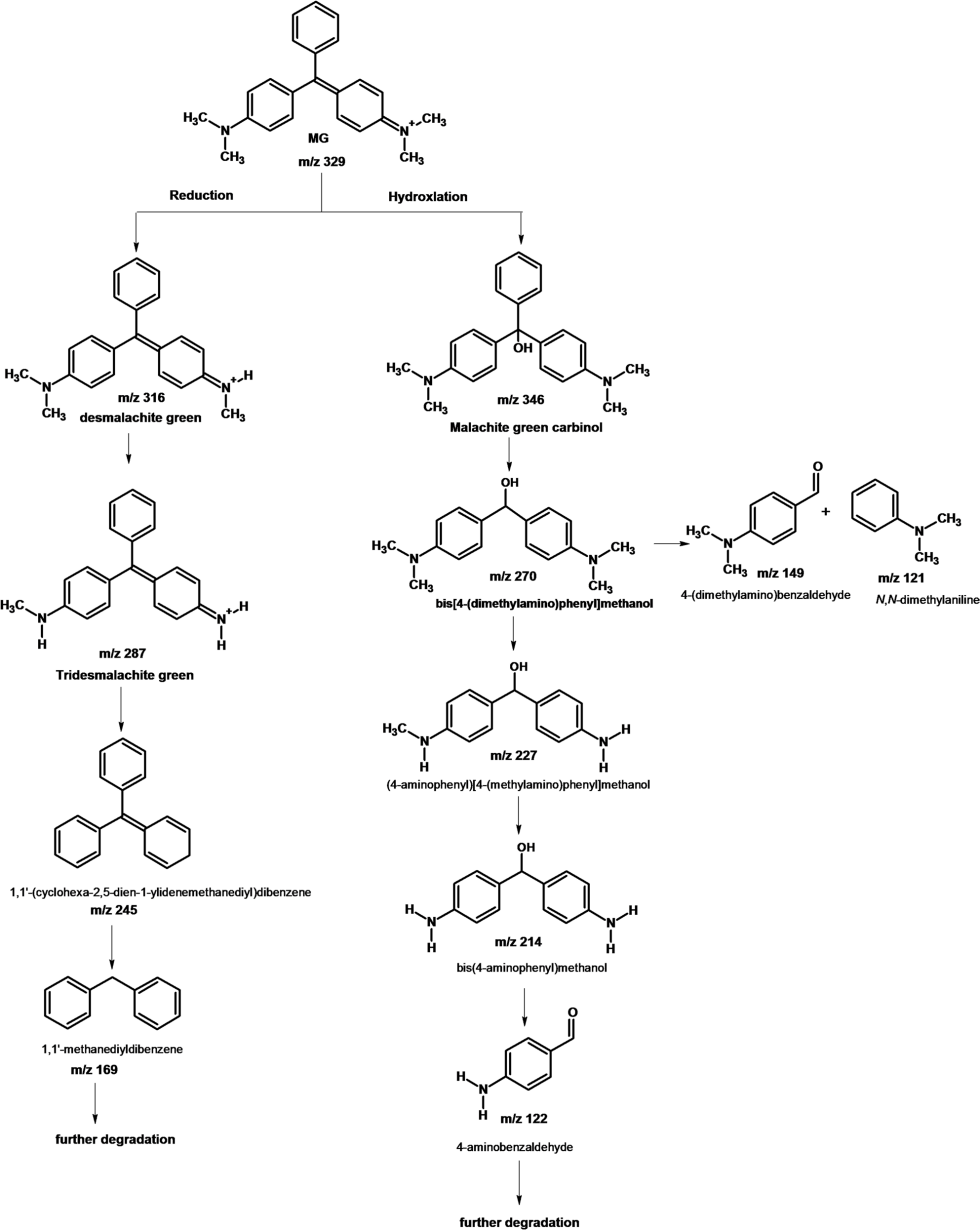
Suggested pathway for the biodegradation of MG by a bacterial consortium

Figure 8 displays the degradation products of MG that were identified using LC–ESI–MS analysis. Des malachite green (m/z 316, Rt 16.81 min), tri desmalachite green (m/z 287, R_t_ 11.87 min), 1,1’-(cyclohexa-2,5-dien-1-ylidenemethanediyl) dibenzene (m/z 316, R_t_ 16.81 min), 1,1’-methanediyldibenzene (m/z 169, R_t_ 9.93 min), malachite green carbinol (m/z 346, R_t_ 10.46 min), bis[4-(dimethylamino) phenyl] methanol (m/z 270, R_t_ 15.91 min), 4-aminophenyl[4-(methylamino)phnyl]methanol (m/z 227,), bis(4-aminophenyl)methanol (m/z 214, R_t_ 14.17 min), and 4-aminobenzaldehydeyde (m/z 31.05 min). Figure 9 shows the two proposed pathways for the biodegradation of MG: either through a sequential process of hydroxylation and demethylation, or indirectly via an oxidative breakdown followed by demethylation. Previous research suggested that the demethylation process, or a reduction reaction followed by demethylation, is the initial step in the biodegradation of MG. *Bacillus subtilis* IF0 13719 and N. coralline produced diaminophenol and ketone of Michler during the degradation of crystal violet. They observed similar degradation processes of triphenylmethane dye by other bacteria [1, 65, 66]. Additionally, *Exiguobacterium* sp. MG2 converted MG to leucomalachite through hydrogenation, leading to the formation of bis[4-(dimethylamino)phenyl] methanol, 3-dimethylamino-phenol, and benzaldehyde [67]. Leucomalachite green, 1,3-benzenedicarboxylic acid, ditertbutyl 2-phenylethoxy silane, bis-2-ethylhexyl ester, 1,4-benzenedicarboxylic acid, 1,2-benzenedicarboxylic acid, di-n-octyl phthalate, and dioctyl ester were detected in the GC-MS and HPLC analysis of the degradation products of MG by *Streptomyces exfoliates* [68]. Additionally, the degradation of MG by *Pseudomonas veronii* led to the identification of five intermediate byproducts [69]. These byproducts included benzaldehyde, hydroquinone, 4-dimethylamino-benzophenone, leucomalachite green, and 4-dimethylaminophenol.

The degradation of MG might due to bacterial consortium enzyme systems which had oxidative and reductive properties play a major role in the transformation. According to numerous reports, laccase is an oxidase that catalyzes the breakdown of MG by oxidizing phenolic and aromatic chemicals [1, 65]. Targeting azo linkages frequently present in dyes, azoreductase is a reductive enzyme that aids in the reduction of MG to less hazardous metabolites, frequently operating in anaerobic environments [66, 67]. Enzymes called dehydrogenase catalyze oxidation-reduction processes that promote mineralization in microbial pathways by further breaking down MG intermediates [1, 67]. When these enzyme systems work together, they may effectively biodegrade MG through oxidation, reduction, and demethylation pathways, which leads to detoxification and color removal. *Pseudomonas* strains with high MG biodegradation efficiency are highlighted in the studies by **Pandey et al.** [1] **and El-Bendary et al.** [65], further supporting the role of these enzymes in the degradation process. The encouraging potential of microbial bioremediation techniques against MG pollution is supported by this enzymatic synergy. **Bibi et al.** [2] identified *Bacillus pacificus* ROC1 has the ability to detoxify MG. Our study supports these findings by showing high degradation efficiency. We have advanced the application of bacterial consortium, validated a hybrid model experimentally, optimized degradation through advanced design methodologies, and analyzed degradation pathways. This provides comprehensive insights into MG bioremediation as presented in Table 6.

**Table 6:** Compound, m/z, retention time (min), formula, and role/intermediate that relate to the degradation pathways of malachite green as described in the literature

Compound	MZ	Retention Time (min)	Formula	Role/Intermediate	Organism	Literature Reference
Malachite Green (MG)	329	Varies (e.g., ~ 12–22)	C_23_H_25_N_2_	Initial compound	*E. sp. MG2*, *P. veronii* JW3-6	[68, 69]
Desmalachite green	316	Varies (e.g., ~ 16.81)	C_22_H_23_N_2_	Intermediate (*N*-demethylation)	*Exiguobacterium sp. MG2*	[65, 67]
Tridesmalachite green	287	Varies (e.g., ~ 11.87)	C_20_H_19_N_2_	Intermediate (*N*,* N*-demethylation)	*Exiguobacterium sp. MG2*	[65, 67]
1,1’-(cyclohexa-2,5-dien-1-ylidenemethanediyl) dibenzene	245	Varies (e.g., ~ 1.05)	C_19_H_16_	Intermediate (amine elimination)	*E. sp. MG2*, *P. veronii* JW3-6	[68, 69]
1,1’-methane- diyldibenzene	169	~ 9.93	C_13_H_12_	Intermediate (Benzene ring cleavage product)	*Pseudomonas veronii* JW3-6	[69]
Malachite green carbinol	346	Varies (e.g., ~ 10.46)	C_23_H_26_N_2_O	Intermediate (hydroxylation)	*Pseudomonas aeruginosa* ED24	[1, 65, 66]
bis[4-(dimethyl amino) phenyl] methanol	270	~ 15.91	C_17_H_22_N_2_O	Intermediate Benzene ring cleavage product	*Bacillus subtilis* and *N. coralline*	[65–67]
4-aminophenyl [4(methylamino) phenyl]methanol	227	Varies (e.g., ~ 12.85)	C_14_H_16_N_2_O	Intermediate (*N*,* N*-demethylation)	*Exiguobacterium sp. MG2*	[65, 67]
bis(4-aminophenyl)methanol	214	~ 14.7	C_13_H_14_N_2_O	Intermediate (*N*-demethylation)	*Exiguobacterium sp. MG2*	[67]
4-amino benzaldehydeyde	122	~ 31.05	C_7_H_7_NO	Intermediate (aniline ring cleavage product)	*Pseudomonas veronii* JW3-6	[69]

### Bacteriotoxic effects

The microbial toxicity of treated and untreated MG dye was evaluated against various bacterial strains, including *Pseudomonas aeruginosa, Serratia marcescens, Escherichia coli, Salmonella typhimurium, Enterobacter aerogenes, Staphylococcus aureus, and Bacillus subtilis*. The treated MG dye showed no growth inhibition around wells containing the degraded dye solution, confirming its non-toxic nature to these microorganisms (Table 7). In contrast, the untreated MG dye inhibited the growth of all tested bacterial strains, demonstrating its toxic effect. This suggests that the bacterial consortium can effectively detoxify MG dye, producing metabolites that are non-toxic to bacteria. In contrast, the original dye at a concentration of 1.0 mg/mL exhibits antimicrobial activity due to its toxicity. These findings highlight the potential of microbial treatment in reducing the toxicity of industrial dyes, making effluent safer for the environment and microbial life.

**Table 7:** Bacterial toxicity of treated and untreated malachite green (MG)

Pathogenic bacteria	Inhibition zone (mm)	
	Untreated MG (control)	Treated MG
*Pseudomonas aeruginosa*	27.50 ± 3.53^a^	-
*Serratia marcescens*	0	-
*Escherichia coli*	27.00 ± 1.41^a^	-
*Salmonella typhimurium*	27.00 ± 2.82^a^	-
*Enterobacter aerogenes*	26.00 ± 1.42^a^	-
*Staphylococcus aureus*	26.00 ± 2.82^a^	-
*Bacillus subtilis*	23.50 ± 0.71^a^	-
F-value	42.227***	-

### Molecular differentiation using ISSR technique

**Fig. 10:**
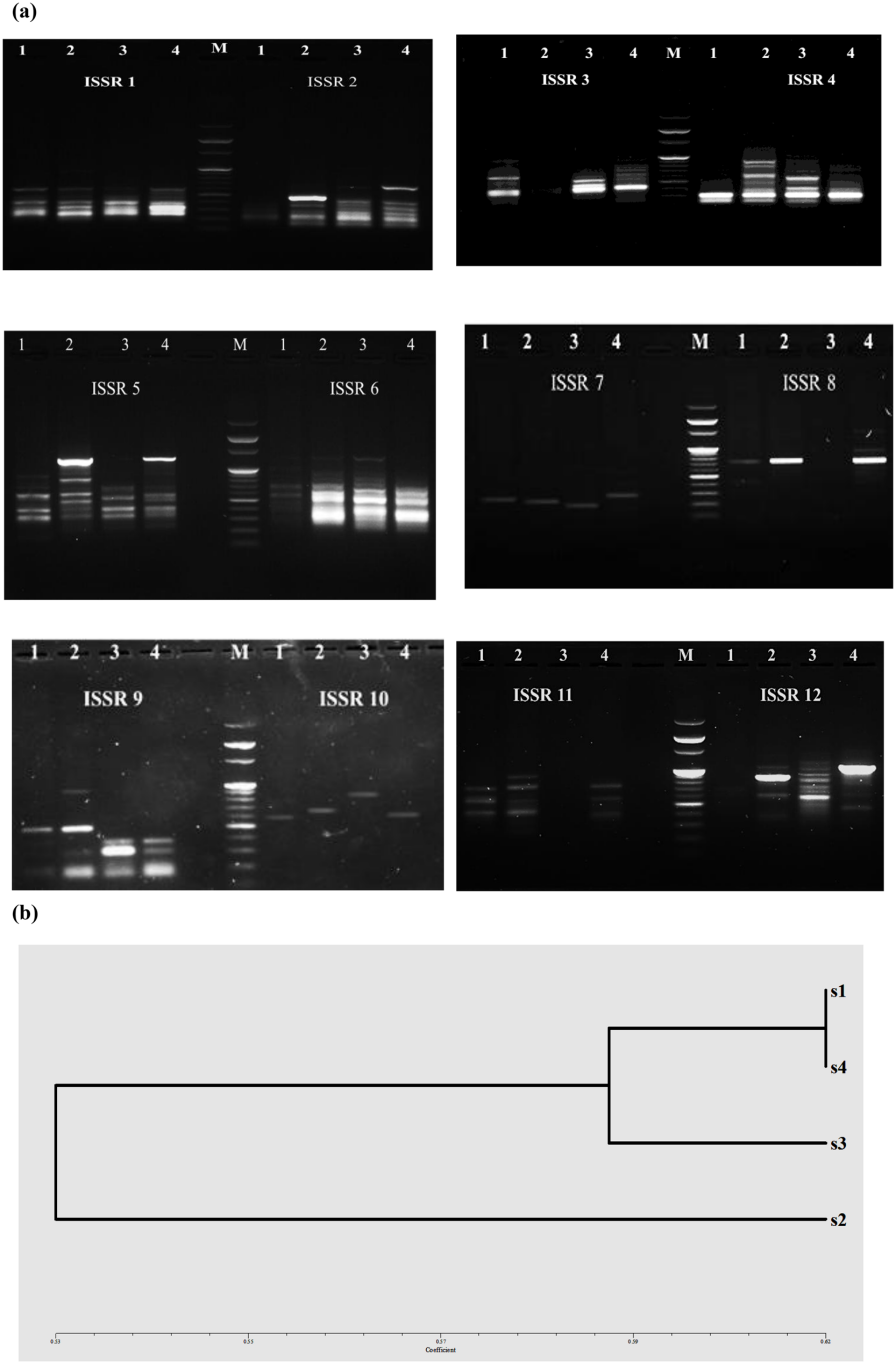
Molecular differentiation between bacterial strains. (**a**) Agarose-gel electrophoresis of ISSR-PCR products from the four bacterial strains: 1, *Azotobacter chroococcum*; 2, *Azotobacter salinestris*; 3, *Stenotrophomonas maltophilia*; 4, *Sphingomonas kyeonggiensis*. M, 100-bp DNA ladder (Enzynomics). (**b**) A genetic dendrogram illustrates the fingerprint profiles and relationships among the bacterial strains. S1–S4 correspond to: **S1**, *Azotobacter chroococcum*; **S2**, *Azotobacter salinestris*; **S3**, *Stenotrophomonas maltophilia*; **S4**, *S. kyeonggiensis*

The ISSR PCR technique was used to detect the genetic variability between the four bacterial strains under study. In this study, all twelve ISSR primers produced scorable and reproducible banding patterns (representative of these PCR products are shown in Fig. 10a. A total of 67 full bands were scored from the amplified products. All primers gave monomorphic bands except primers (ISSR-5, ISSR-7, ISSR-8, ISSR-10, ISSR-11, and ISSR-12). Polymorphism is informative to demonstrate the genetic diversity where these unique bands significantly mark individual species, also it distinguishes an individual from its population. As a primer’s discriminatory power is related to its amplified polymorphic bands relative to that produced by the rest of all primers. Primer ISSR-12 reflected high discriminatory power as compared with other primers; it generated nine unique bands. While the maximum number of monomorphic bands was amplified by primer ISSR-1 (see Fig. 10a and Table 8a). As shown in Fig. 10b and Table 8b, the phylogenetic tree illustrates two distinct clades. The first clade includes *A. chroococcum*, *S. kyeonggiensis*, and *S. maltophilia*. The strain of *S. kyeonggiensis* scores 62% similarity with both *A. chroococcum* and *S. maltophilia*. The second clade comprises *A. salinestris* which shares 0.52 similarity with *S. kyeonggiensis*. In this study, PCR-ISS technique was employed to demonstrate the genetic diversity between the bacterial strains as ISSR markers are highly polymorphic and are useful in studies on genetic diversity, phylogeny, gene tagging, genome mapping, and evolutionary biology [70]. Our findings revealed that although strains S1 and S2 both belong to the genus *Azotobacter*, they were placed in different clades. In contrast, *A. chroococcum*, *S. kyeonggiensis*, and *S. maltophilia* clustered together in a single clade. This suggests that horizontal gene transfer (HGT) or lateral gene transfer (LGT) and homologous recombination (HR) both serve as principal evolutionary forces in bacteria specially among different genera, causing genetic evolution and DNA rearrangement [71].

**Table 8:** Numbers of DNA fragments amplified by the twelve ISSR primers in bacterial strains (a), and similarity matrix among bacterial strains (b)

Primers	Monomorphic bands	Polymorphic bands		No. of total bands	Polymorphism%	Range of molecular weight (bp)
		Unique	Non-unique			
**(a)**						
ISSR-1	4	1	0	5	0.20	200–900
ISSR-2	3	1	3	7	0.57	150–600
ISSR-3	1	1	1	3	0.67	220–900
ISSR-4	2	4	1	7	0.71	220–900
ISSR-5	0	1	5	6	1.00	200–1200
ISSR-6	2	1	3	6	0.67	250–1100
ISSR-7	0	2	1	3	1.00	20–250
ISSR-8	0	3	1	4	1.00	700–1100
ISSR-9	1	1	3	5	0.80	200–900
ISSR-10	0	3	0	3	1.00	600–750
ISSR-11	0	2	3	5	1.00	250–900
ISSR-12	0	9	0	9	1.00	350–1100
**(b)**						
	**S1**	**S2**	**S3**	**S4**		
S1	1.00					
S2	0.57	1.00				
S3	0.57	0.51	1.00			
S4	0.62	0.52	0.62	1.00		

**S1**: *Azotobacter chroococcum*, **S2**: *Azotobacter salinestris*, **S3**: *Stenotrophomonas maltophilia*, **S4**: *Sphingomonas kyeonggiensis*

Sequences amplified by ISSR-PCR can be used for DNA fingerprinting. Since an ISSR may be a conserved region, this technique is useful for distinguishing individuals. Other studies have demonstrated the efficiency of PCR-ISSR to molecularly characterize the wild type of Bacillus cereus and its mutants [72]. In another study, the ISSR molecular markers were used to evaluate genetic diversity assessment in microbial diversity [73]. Also, seven ISSR to study the genetic diversity of three different *Bacillus* isolates used to enhance sustainable agricultural productivity, as they promote a consortium in the drought-tolerant plant [74].

In summary, the proposed hybrid framework, which combines a dynamic RSM baseline with ML models alongside the developed bacterial consortium, is scalable to experimental, pilot, and full industrial wastewater-treatment applications for MG dye removal and can be implemented as a low-chemical, biologically based option. By reducing the number of trial-and-error experiments required for process optimization, the hybrid strategy lowers energy consumption, experimental time, labour, overall cost, and the associated carbon footprint, thereby aligning with global sustainability goals and representing a “green” approach to industrial pollutant treatment. Reduced toxic dye discharge and more sustainable textile effluent management are two obvious environmental effects of this. Future research will concentrate on evaluating the long-term stability of consortium, conducting a techno-economic analysis for full-scale deployment, and validating the model across a larger dataset and operating conditions. Once its performance has been confirmed on larger datasets, the hybrid model can be deployed as a user-friendly, interactive web application to support decision-making in real-world operations.

## Conclusion

Microbial consortia can effectively metabolize MG as a single biocatalyst unit, demonstrating their broad catabolic capacity and potential for environmental cleanup. In this study, a highly potent bacterial consortium could degrade 100 mg/L of MG. The BBD analysis revealed that the optimal conditions for MG biodecolorization are a concentration of 100 mg/L, 7% inoculum size, and a 72-h incubation period, resulting in a 115.63% increase in MG removal when comparing with MD. The study introduces a hybrid modeling approach that combines a dynamic model with residual machine learning regressors to predict MG dye decolorization efficiency. The dynamic model achieved high accuracy, while linear residual learners enhanced performance. Nonlinear ensemble regressors were less stable. BBD can be a valuable tool for process optimization in bioremediation, and the formulated consortium can effectively treat industrial effluents. Future research should explore advanced hybrid architecture, expand the dataset, and address uncertainty quantification. This approach bridges the gap between ML adaptability and mechanistic understanding.

## Supplementary Information

Below is the link to the electronic supplementary material.

Supplementary Material 1

## Data Availability

All data generated or analyzed during this study are included in this published article.
