# Supplementary material for: Residual-hybrid dynamic response-surface/machine-learning framework for predicting malachite green removal and biodegradation by a bacterial consortium
Source: J Biol Eng. 2026 Apr 2;20:64. doi: 10.1186/s13036-026-00663-8 (PMC13063629; doi:10.1186/s13036-026-00663-8)
Supplement: Supplementary file 1 — Supplementary Material 1 [file 13036_2026_663_MOESM1_ESM.docx]

**Supplementary information to the article “Residual-hybrid dynamic response-surface/machine-learning framework for predicting malachite green removal and biodegradation by a bacterial consortium” (Sakr et al.)**

## **Table S1**

## Proposed summary of machine-learning models used in the residual-learning framework

| **Model** | **Category** | **Brief description** | **Reference** |
| --- | --- | --- | --- |
| Ridge | Linear | Linear regression with L2 regularization reduces overfitting and handles multicollinearity. | Hoerl & Kennard, (1970). |
| Lasso | Linear | Linear regression with L1 regularization performs variable selection and shrinkage. | Tibshirani, R. (1996). |
| ElasticNet | Linear | Combines L1 and L2 penalties, balancing variable selection and stability. | Zou & Hastie (2005) |
| KNN | Nonlinear | Instance-based learners predict using a weighted average of nearest neighbors. | Cover & Hart (1967). |
| Random Forest | Nonlinear (Ensemble) | Bagging of decision trees reduces variance and improves robustness. | Breiman, L. (2001) |
| Gradient Boosting | Nonlinear (Ensemble) | Sequential boosting of trees using gradient optimization. | Friedman, J. H. (2001). |
| XGBoost | Nonlinear (Ensemble) | Optimized gradient boosting with regularization and scalability. | Chen, T., & Guestrin, C. (2016, |
| CatBoost | Nonlinear (Ensemble) | Gradient boosting with efficient handling of categorical variables. | Dorogush, et al. (2018). |

| **Input:**  • C0[i] – initial MG concentration for experiment i  • Inoc[i] – inoculum amount  • Time[i] – incubation time  • y_dyn[i] – dynamic-model prediction (Design-Expert)  • y_actual[i]– experimental MG removal  • N = 17 – number of experimental runs  **1. Compute dynamic-model residuals**  For each experiment i = 1,…,N:  Residual[i] = y_actual[i] − y_dyn[i]  **2. Construct input features for residual learning**  For each experiment i = 1,…,N:  X[i] = ( C0[i], Inoc[i], Time[i], y_dyn[i] )  Set target vector:  y_res = Residual  **3. Residual-model selection using LOOCV**  For each candidate model M ∈ {RidgeCV, KNN, Gradient Boosting, Random Forest}:  For k = 1,…,N:  – Train M on all samples except k using (X, y_res)  – Predict residual for the left-out sample:  r_pred[k] = M( X[k] )  End for  – Compute performance metrics for model M:  R²_M, RMSE_M, MAE_M  End for  **4. Train the final residual learner**  – Select M* with the lowest RMSE_M (and highest R²_M)  – Retrain M* on all N samples to obtain g(·)  **5. Generate hybrid predictions**  For any experiment i:  – Predict residual: r̂[i] = g( X[i] )  – Hybrid prediction: y_hybrid[i] = y_dyn[i] + r̂[i]  **Output:**  • Final residual learner g(·)  • Hybrid predictions y_hybrid[i]  • Performance metrics (R², RMSE, MAE, MAPE, sMAPE) |
| --- |

**Figure S1**: Algorithm of Hybrid Dynamic–Residual Learning Procedure

**Table S2.**

Nucleotide sequences of ISSR primers used in this study.

| **Primer** | **Sequence** |
| --- | --- |
| **ISSR- 1** | 5'-AGAGAGAGAGAGAGAGYC-3' |
| **ISSR- 2** | 5'-AGAGAGAGAGAGAGAGYG-3' |
| **ISSR- 3** | 5'-ACACACACACACACACYT-3' |
| **ISSR- 4** | 5'-ACACACACACACACACYG-3' |
| **ISSR- 5** | 5'-ACACACACACACACACYA-3' |
| **ISSR- 6** | 5'-ACACACACACACACACYC-3' |
| **ISSR- 7** | 5'-AGAGAGAGAGAGAGAGYT-3' |
| **ISSR- 8** | 5'-CTCCTCCTCCTCCTCTT-3' |
| **ISSR- 9** | 5'-CTCTCTCTCTCTCTCTRG-3' |
| **ISSR- 10** | 5'-TCTCTCTCTCTCTCTCA-3' |
| **ISSR- 11** | 5'-HVHCACACACACACACAT-3' |
| **ISSR- 12** | 5'-HVHTCCTCCTCCTCCTCC-3' |

**Table S 3:**

Regression coefficients with 95% confidence intervals for MD Model.

| **Component** | **Estimate Coefficient** | **Standard Error (SE)** | **95% Confidence Interval** |
| --- | --- | --- | --- |
| A-X1 | 68.65 | 1 | (66.2 , 71.1) |
| B-X2 | 33.03 | 1 | (30.58, 35.48) |
| C-X3 | 67.68 | 1 | (65.23, 70.12) |
| D-X4 | 29.52 | 1 | (27.07, 31.97) |
| AB | 106.54 | 6.33 | (91.06, 122.02) |
| AC | 61.88 | 6.32 | (46.42, 77.34) |
| AD | 61.22 | 6.32 | (45.75, 76.68) |
| BC | 97.82 | 6.32 | (82.35, 113.29) |
| BD | 9.51 | 6.32 | (-5.96, 24.97) |
| CD | 24.91 | 4.9 | (12.91, 36.92) |
| ABC | -644.33 | 185.55 | (-1098.35, -190.32) |
| ABD | -1127.57 | 149.08 | (-1492.36, -762.77) |
| ACD | -281.85 | 178.54 | (-718.71, 155.01) |
| BCD | 1733.48 | 179.99 | (1293.07, 2173.89) |

**X1**: *A. chroococcum* 6FE; **X2**: *A. salinestris* 9FE; **X3**: *S. maltophilia*; **X4**: *S. kyeonggiensis*

| **(a)** | **(b)** |
| --- | --- |
|  |  |
| **(c)** | **(d)** |
|  |  |
| **(e)** | |
|  | |

**Figure S2.** Model diagnostic plots: (a) normal probability plot of the internally studentized residuals; (b) residuals vs. predicted values; (c) residuals vs. run order; (d) predicted vs. actual values; and (e) Box–Cox power transformation plot.

**Table S4**

Regression Coefficients with 95% Confidence Intervals for BBD Model.

| **Component** | **Estimate Coefficient** | **Standard Error (SE)** | **95% Confidence Interval** |
| --- | --- | --- | --- |
| Intercept | 83.1 | 1.83 | (78.78, 87.42) |
| A-A | -28.62 | 1.44 | (-32.03, -25.2) |
| B-B | 4.94 | 1.44 | (1.52, 8.35) |
| C-C | 8.56 | 1.44 | (5.15, 11.98) |
| AB | 13.11 | 2.04 | (8.28, 17.94) |
| AC | 3.99 | 2.04 | (-0.84, 8.82) |
| BC | 0.44 | 2.04 | (-4.39, 5.27) |
| A^^2^ | -21.01 | 1.99 | (-25.72, -16.3) |
| B^^2^ | -0.42 | 1.99 | (-5.13, 4.29) |
| C^^2^ | 1.28 | 1.99 | (-3.42, 5.99) |

A: Initial dye concentration, B: Amount of consortia inoculum, C: Incubation time

| **(a)** |
| --- |
| **** |
| **(b)** |
| **** |

**Figure S3.** Diagnostic plots for the **Box–Behnken** design: **(a)** normal probability plot of the internally studentized residuals; **(b)** predicted **vs.** actual MG decolorization.

| **(a)** |
| --- |
| 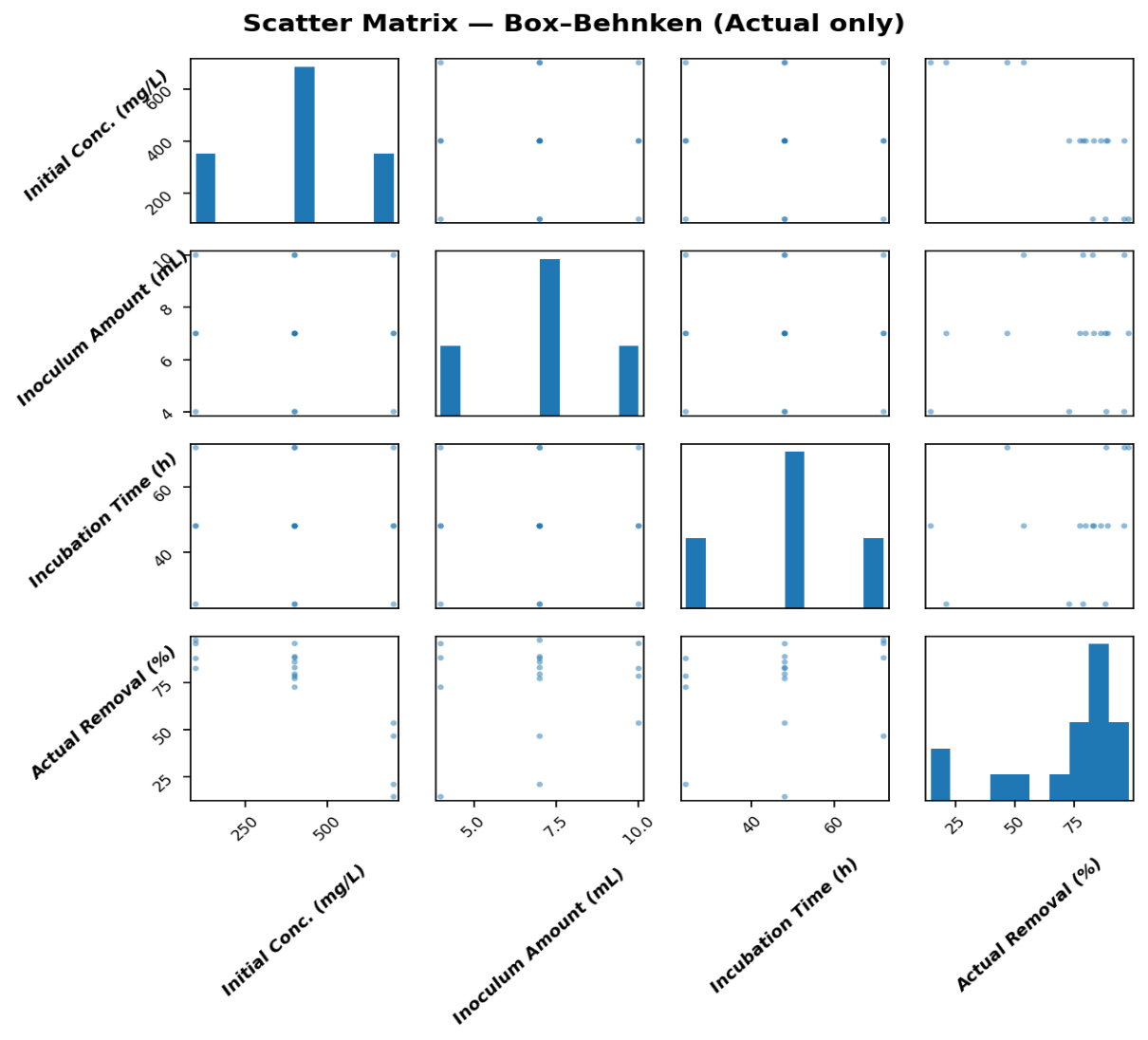 |
| (b) |
| 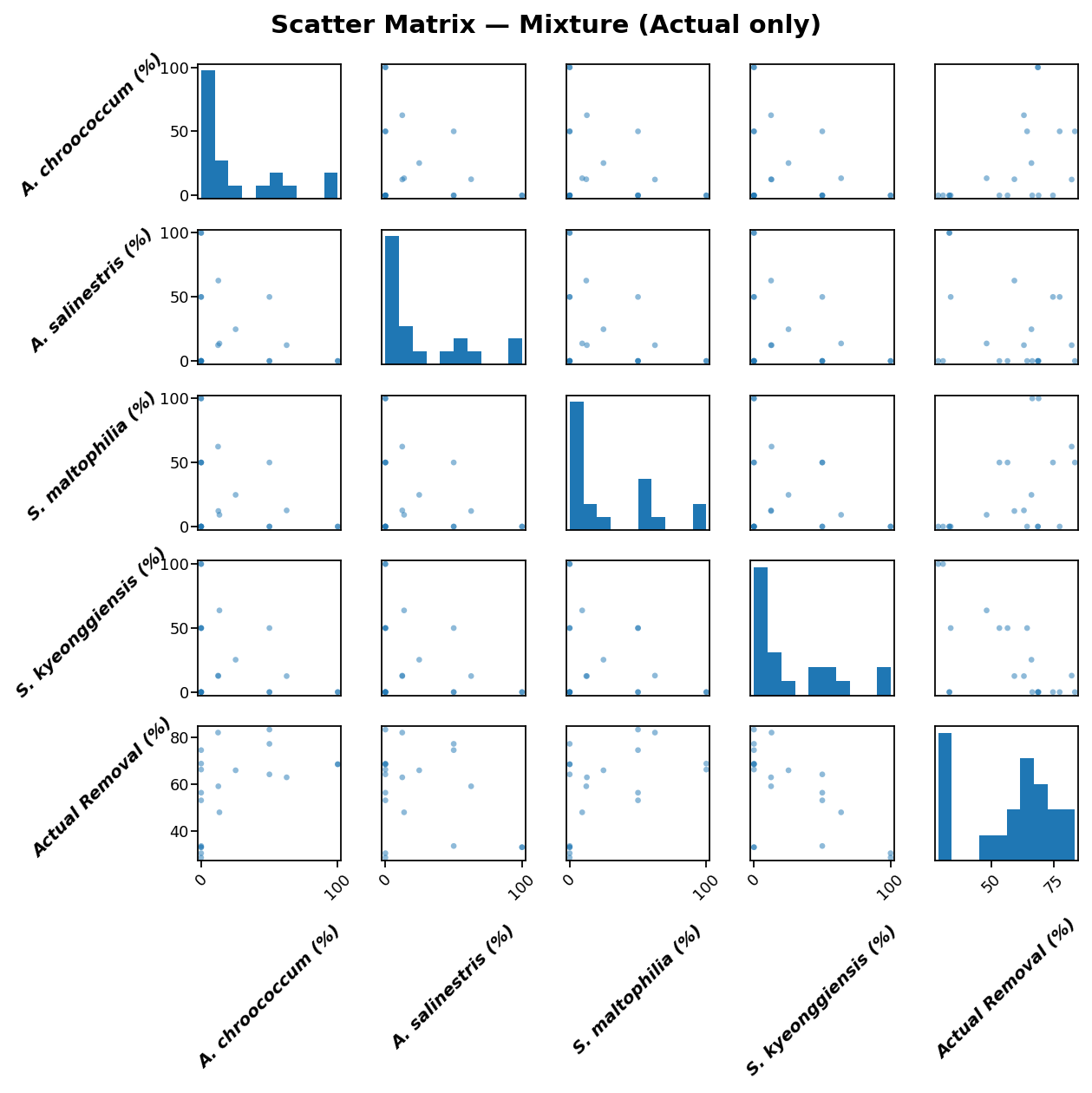 |

**Figure S4.** Scatterplot matrices for the Box–Behnken (a) and Mixture (b) experimental designs, showing pairwise relationships among the design variables and actual MG removal.

| **(a)**  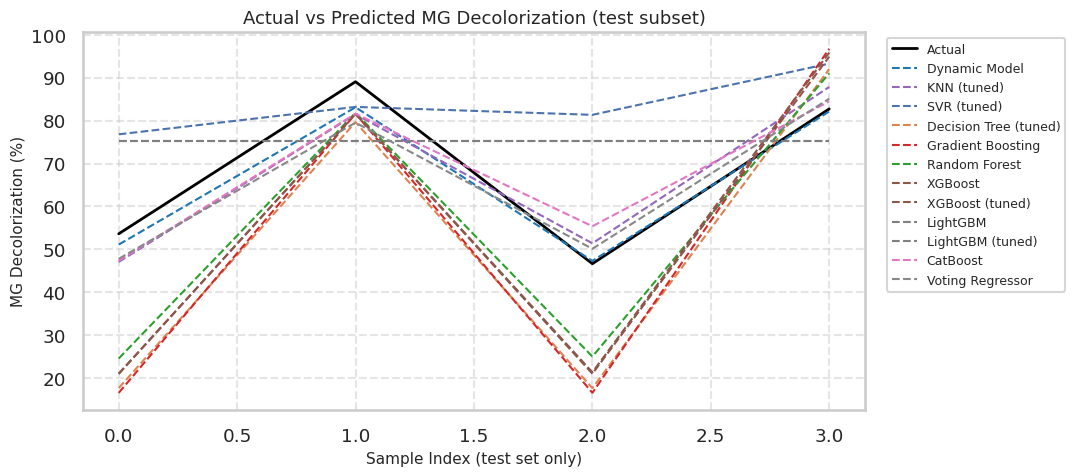 |
| --- |
| **(b)** |
| **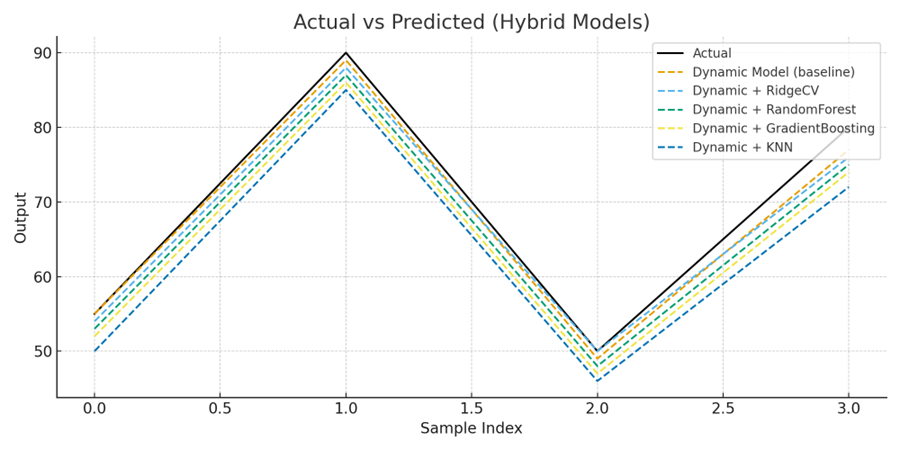** |

**Figure S5.** Actual vs. predicted MG decolorization (%): **(a)** Baseline Dynamic Model (RSM-based dynamic response-surface baseline) and standalone machine-learning regressors evaluated on the 80/20 train–test split (n = 4): Dynamic Model (R² = 0.968, RMSE = 3.26), KNN (R² = 0.887, RMSE = 6.11), CatBoost (R² = 0.868, RMSE = 6.60), Random Forest (R² = –0.084, RMSE = 18.94), SVR (R² = –0.426, RMSE = 21.71),

Decision Tree (R² = –0.745, RMSE = 24.02), Gradient Boosting (R² = –0.913, RMSE = 25.15), XGBoost (R² = –0.454, RMSE = 21.93), LightGBM (R² = –0.162, RMSE = 19.61). **(b)** Hybrid residual models using the Dynamic Model (RSM baseline) as a structured baseline, evaluated using **LOOCV (N = 17)**: Dynamic Model (R² = 0.988, RMSE = 2.62), Dynamic + RidgeCV (R² = 0.987, RMSE = 2.78), Dynamic + Random Forest (R² = 0.981, RMSE = 3.25), Dynamic + Gradient Boosting (R² = 0.982, RMSE = 3.23), Dynamic + KNN (R² = 0.975, RMSE = 3.88).

***RSM****, response-surface methodology;* ***KNN****, k-nearest neighbors;* ***SVR****, support vector regression;* ***RF****, random forest;* ***GB****, gradient boosting;* ***XGBoost****, extreme gradient boosting;* ***LightGBM****, Light Gradient Boosting Machine;* ***LOOCV****, leave-one-out cross-validation;* ***RMSE****, root-mean-square error.*

| 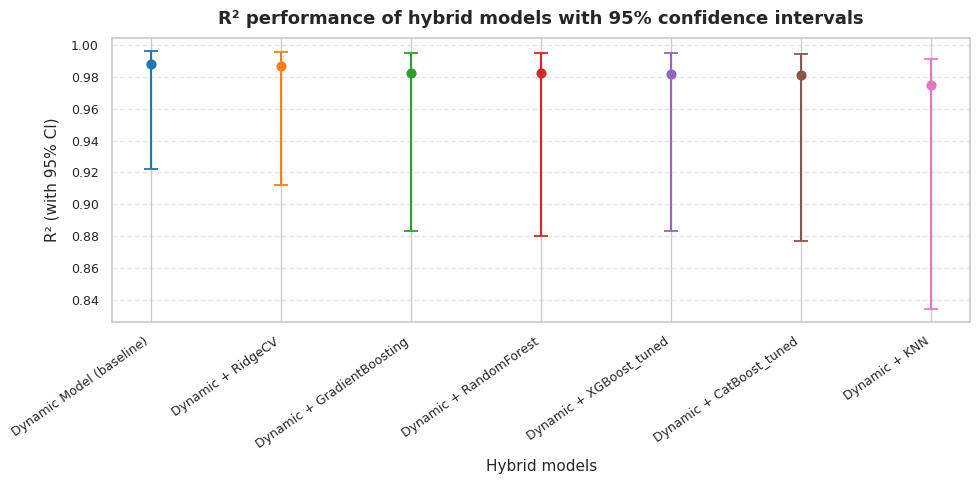 |
| --- |
| 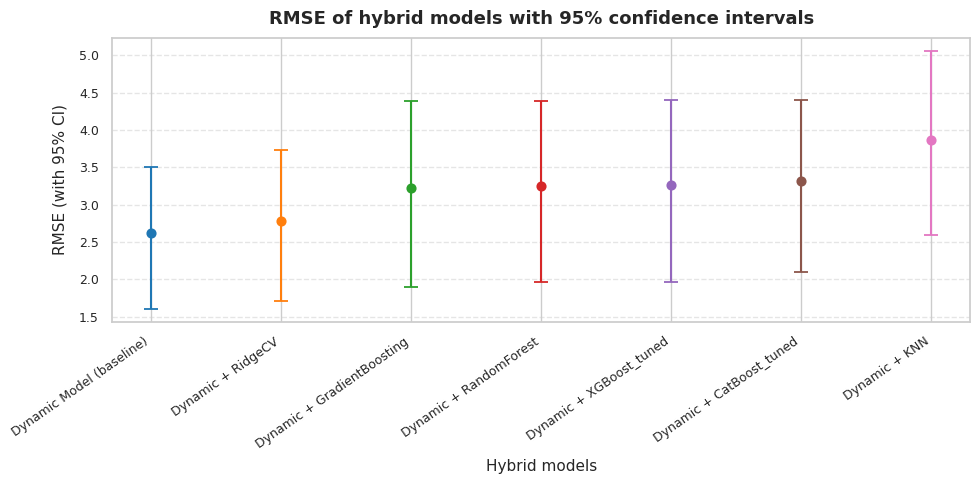 |

**Figure S6**. Performance comparison of the Dynamic RSM baseline and hybrid Dynamic + ML models (Dynamic + RidgeCV, Dynamic + Gradient Boosting, Dynamic + Random Forest, Dynamic + XGBoost, Dynamic + CatBoost, and Dynamic + KNN) for MG decolorization.
(a) Coefficient of determination (R²) values with 95% confidence intervals.
(b) Root mean square error (RMSE %) values with 95% confidence intervals.
***Points*** *indicate mean performance metrics, and vertical bars represent their corresponding 95% confidence ranges.*
